# Factors Associated With Psychological Distress in Health-Care Workers During an Infectious Disease Outbreak: A Rapid Systematic Review of the Evidence

**DOI:** 10.3389/fpsyt.2020.589545

**Published:** 2021-01-28

**Authors:** Fuschia M. Sirois, Janine Owens

**Affiliations:** ^1^Department of Psychology, University of Sheffield, Sheffield, United Kingdom; ^2^School of Clinical Dentistry, University of Sheffield, Sheffield, United Kingdom

**Keywords:** COVID-19, health-care workers, psychological distress, risk factors, resilience, anxiety, stress, depression

## Abstract

**Objective:** Health-care workers (HCW) are at risk for psychological distress during an infectious disease outbreak, such as the coronavirus pandemic, due to the demands of dealing with a public health emergency. This rapid systematic review examined the factors associated with psychological distress among HCW during an outbreak.

**Method:** We systematically reviewed literature on the factors associated with psychological distress (demographic characteristics, occupational, social, psychological, and infection-related factors) in HCW during an outbreak (COVID-19, SARS, MERS, H1N1, H7N9, and Ebola). Four electronic databases were searched (2000 to 15 November 2020) for relevant peer-reviewed research according to a pre-registered protocol. A narrative synthesis was conducted to identify fixed, modifiable, and infection-related factors linked to distress and psychiatric morbidity.

**Results:** From the 4,621 records identified, 138 with data from 143,246 HCW in 139 studies were included. All but two studies were cross-sectional. The majority of the studies were conducted during COVID-19 (*k* = 107, *N* = 34,334) and SARS (*k* = 21, *N* = 18,096). Consistent evidence indicated that being female, a nurse, experiencing stigma, maladaptive coping, having contact or risk of contact with infected patients, and experiencing quarantine, were risk factors for psychological distress among HCW. Personal and organizational social support, perceiving control, positive work attitudes, sufficient information about the outbreak and proper protection, training, and resources, were associated with less psychological distress.

**Conclusions:** This review highlights the key factors to the identify HCW who are most at risk for psychological distress during an outbreak and modifying factors to reduce distress and improve resilience. Recommendations are that HCW at risk for increased distress receive early interventions and ongoing monitoring because there is evidence that HCW distress can persist for up to 3 years after an outbreak. Further research needs to track the associations of risk and resilience factors with distress over time and the extent to which certain factors are inter-related and contribute to sustained or transient distress.

## Introduction

Several outbreaks of viral diseases have posed significant public health threats since 2000. These include SARS, H1N1, H7N9, MERS, EBOLA, and more recently, COVID-19 (see Supplementary Table 1). Such outbreaks place a serious strain on the health-care systems that try to contain and manage them, including health-care workers (HCW) who are at increased risk for nosocomial infections (1). In addition to the threat to their own physical health, HCW can experience psychological distress as a collateral cost of the risk of infection and the demands of dealing with a public health emergency (2).

Psychological distress refers to a state of emotional suffering, resulting from being exposed to a stressful event that poses a threat to one's physical or mental health (3). Inability to cope effectively with the stressor results in psychological distress that can manifest as a range of adverse mental health and psychiatric outcomes including depression, anxiety, acute stress, post-traumatic stress, burnout, and psychiatric morbidity. Although psychological distress is often viewed as a transient state that negatively impacts day-to-day and social functioning, it can persist and have longer-term negative effects on mental health (4).

Under normal circumstances, work-related psychological distress in HCW is associated with several short and long-term adverse outcomes. Psychological distress is linked to adverse occupational outcomes including include decreased quality of patient care (5), irritability with colleagues (6), cognitive impairments that negatively impact patient care (7), and intentions to leave one's job (8). HCW who experience psychological distress are also at risk of experiencing adverse personal outcomes including substance misuse (6), and suicide (9). In the context of an infectious disease outbreak, such consequences may amplify and heighten psychological distress. HCW who reported elevated levels of psychological distress during the COVID-19 outbreak also experienced sleep disturbances (10), poorer physical health (11), and a greater number of physical symptoms, including headaches (12). Similarly, HCW during the SARS outbreak disclosed a greater number of somatic symptoms and sleep problems (13), substance misuse and more days off work (14).

Apart from the immediate and short-term impacts on HCW mental health, there is limited but concerning evidence, that working during an infectious outbreak can have lasting and detrimental psychological effects for HCW. In a study of HCW who worked during the SARS outbreak in China, 10 percent experienced high levels of post-traumatic stress (PTS) symptoms when surveyed 3 years later (15). Similarly, HCW who treated patients during the SARS outbreak in Canada reported significantly higher levels of burnout, psychological distress, and post-traumatic stress compared to HCW in other hospitals that did not treat SARS patients when surveyed 13–26 months after the SARS outbreak (14). Lastly, a study of HCW in Hong Kong during the SARS outbreak found that although the levels of perceived stress did not differ between HCW who worked in high risk and low risk areas initially, 1 year later the stress of the high-risk HCW was significantly increased, and was higher than the stress reported by the low-risk HCW (16). This increased level of stress was also associated with higher levels of depression, anxiety, and post-traumatic stress, indicating a pervasive and sustained negative impact of working during an outbreak on mental health. These findings underscore the importance of understanding the factors that contribute to risk and resilience for psychological distress in HCW.

HCW serve a vital role in treating and managing infected individuals during an infectious disease outbreak such as coronavirus. There is an urgent need to understand the factors that create or heighten risk for distress for HCW and affect their immediate and long-term mental health during the COVID-19 pandemic and other similar outbreaks, as well as those that are protective and may reduce psychological distress. Such knowledge is important for identifying HCW most at risk, and informing strategies and treatments needed to support HCW resilience during and after an outbreak.

This rapid review synthesized the evidence on the factors associated with psychological distress among health-care workers (HCW) during an infectious disease outbreak. The review focused not only on the COVID-19 pandemic, but also on other related coronavirus and influenza outbreaks (SARS, H1N1, H7N9, MERS, and Ebola), to expand the potential evidence base and to increase the potential for the findings to be generalizable across any future infectious disease outbreaks.

This review also introduced a conceptual framework for understanding and classifying the factors that contributed to risk or provided resilience for psychological distress. Based on our early scan of the literature, we grouped factors into three conceptual categories: (1) fixed or unchangeable factors, (2) potentially modifiable factors, and (3) factors related to infection exposure. Fixed factors were viewed as identifying HCW who might be most vulnerable or resilient to distress and, if the former, require extra support and treatment. Socio-demographic factors and other factors related to work role and experience were included in this category. In contrast, modifiable factors were viewed as identifying potential targets for interventions to reduce risk and increase resilience. Social and psychological factors, such as social support, stigma, and psychological resources such as coping styles and personality were included in the modifiable category. Lastly, infection-related factors were those that can directly inform hospital procedures and operating policy regarding ways to address and mitigate risk. Factors related to infection exposure and risk of exposure, and the provision of training, resources, and personal protective equipment (PPE) were included in this category.

The key questions addressed by this review were:

1. What are the risk factors for psychological distress among HCW during an infectious outbreak?
2. What are the factors associated with reduced risk for psychological distress among HCW during an infectious outbreak?

## Methods

Evidence was summarized using a rapid, systematic review approach because of the urgent need to support the mental health of HCW during and after the ongoing novel coronavirus pandemic. Rapid Reviews are a form of systematic review that provide an expedient and useful means of synthesizing the available evidence during times of health crises to inform evidence-based decision making for health policy and practice (17, 18). To accomplish this, rapid reviews take a streamlined approach to systematically reviewing evidence. Modified methods in the current review included: (1) search limited to English language studies; (2) gray literature limited to one search source; (3) no formal critical appraisal of the research.

### Data Sources and Searches

The search strategy for this pre-registered rapid review involved searching Medline, PsycInfo, Web of Science, and the first 10 pages of Google Scholar, as well as hand searching references. Search terms included a combination of terms related to health-care workers (e.g., “physicians,” “nurses”), and distress (e.g., “stress,” “anxiety”). The full search term list is available on PROSPERO (CRD42020178185). We conducted searches in a rolling manner, starting on April 6, 2020, then with updates on June 7, July 2, July 10, July 30, 2020, and November 15, 2020 to capture and integrate the most up-to-date evidence given the ongoing COVID-19 pandemic and the associated rapid release of research.

### Study Selection and Data Extraction

We used a predefined search strategy (see full details on PROSPERO, https://www.crd.york.ac.uk/PROSPERO/; registration ID: CRD42020178185). Studies were included in this Review if they were empirical research; published or accepted for publication in peer-reviewed journals; written in English; included participants who were HCW who worked in a hospital environment during a major infectious outbreak (COVID19, SARS, MERS, H1N1, H7N9, Ebola); had a sample size of >80, and included data on factors associated with psychological distress during an outbreak. One investigator screened citations for potential full-text review, and a second investigator conducted the full-text review of each study for inclusion. Exclusions were verified by the other investigator, and disagreements resolved through discussion. Data was extracted by one investigator, entered into a table, and verified by a second investigator. For studies that included tests for multiple measures of psychological distress, we included the study as reporting a significant association with a particular factor if at least one of the measures of distress were significant.

Although rapid reviews do not always include a formal assessment of study quality and risk for bias (18), a lack of a quality assessment can have important implications for the utility of the results (17). Accordingly, we evaluated the methodological quality of the studies in the review using a tool adapted for the current study. The assessment tool included eleven questions chosen from the Appraisal tool for Cross Sectional Studies, AXIS (19) as being most relevant for the current study, an approach advocated by Quintana (20). Two authors independently rated the quality of the studies using the 11 questions to assess the quality of the study procedures, sampling, and the measures. The assessment yielded a total score that categorized studies as having low (<5), moderate (5–7), or high (8–10) quality. Inter-rater agreement was calculated and assessed using Cohen's Kappa coefficient (21). Discrepancies were resolved through discussion. In addition to the formal quality assessment, we only included studies that reported findings for a sample size of >80, which allows enough power to detect a medium effect size with an alpha of 0.05 (21, 22).

### Data Synthesis and Analysis

We conceptually organized the factors in this Review identified as contributing to or mitigating psychological distress into three broad categories: (1) fixed or unchangeable factors (sociodemographic and occupational factors), (2) potentially modifiable factors (social and psychological factors), and (3) factors related to infection exposure. Evidence was synthesized according to these conceptual categories, with non-significant and contrary findings noted in addition to significant findings to provide a more complete picture of the weight of the evidence for each factor. The balance of evidence for each factor was further presented graphically. We assigned factors within each conceptual category as reflecting either risk or resilience for psychological distress according to logic and theory (e.g., maladaptive coping as risk, adaptive coping as resilience). Factors that could be interpreted as either risk or resilience (e.g., sex, age) were assigned according to how they had been framed in the majority of the research that examined these factors.

## Results

**Figure 1:**
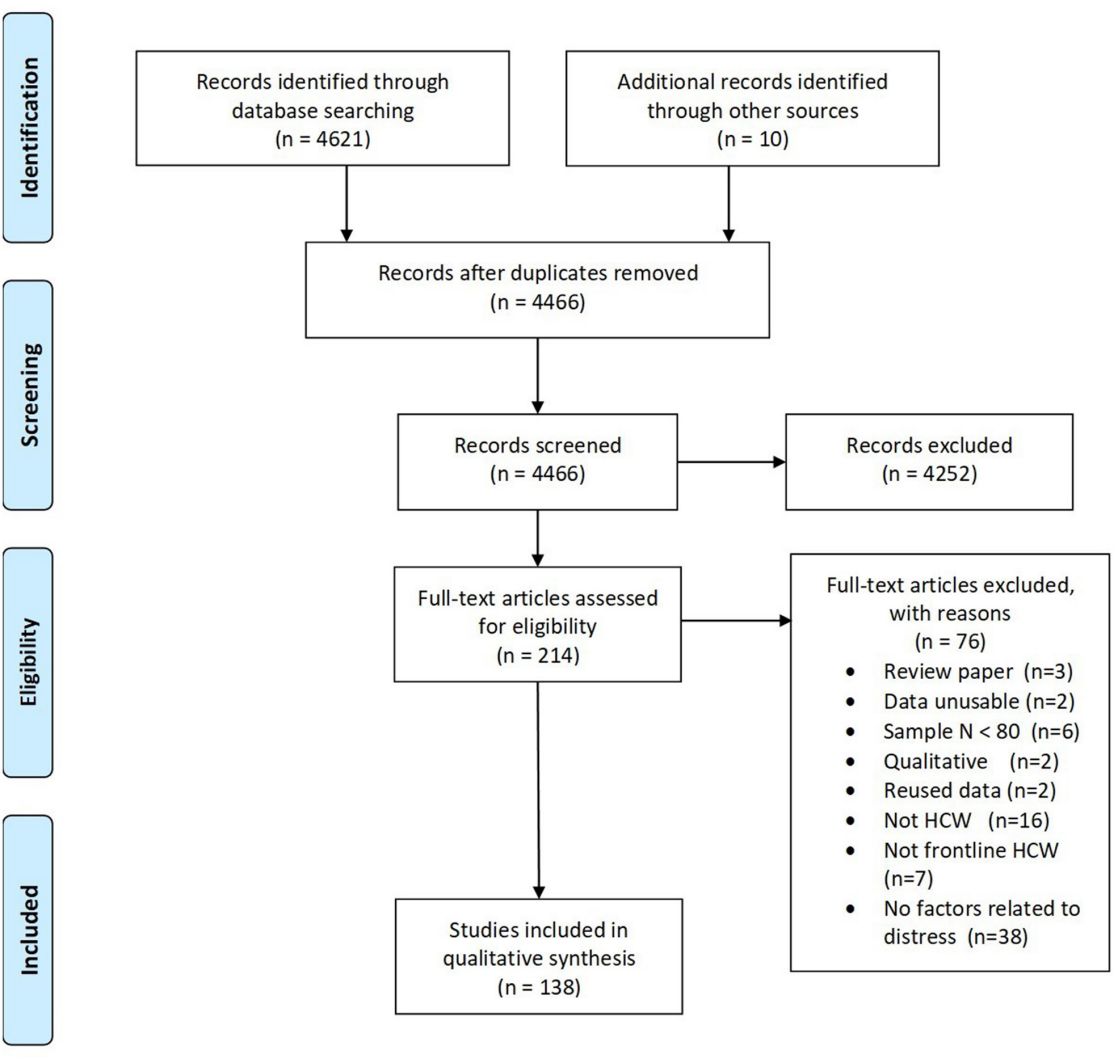
PRISMA flow diagram for literature screening.

**Figure 2:**
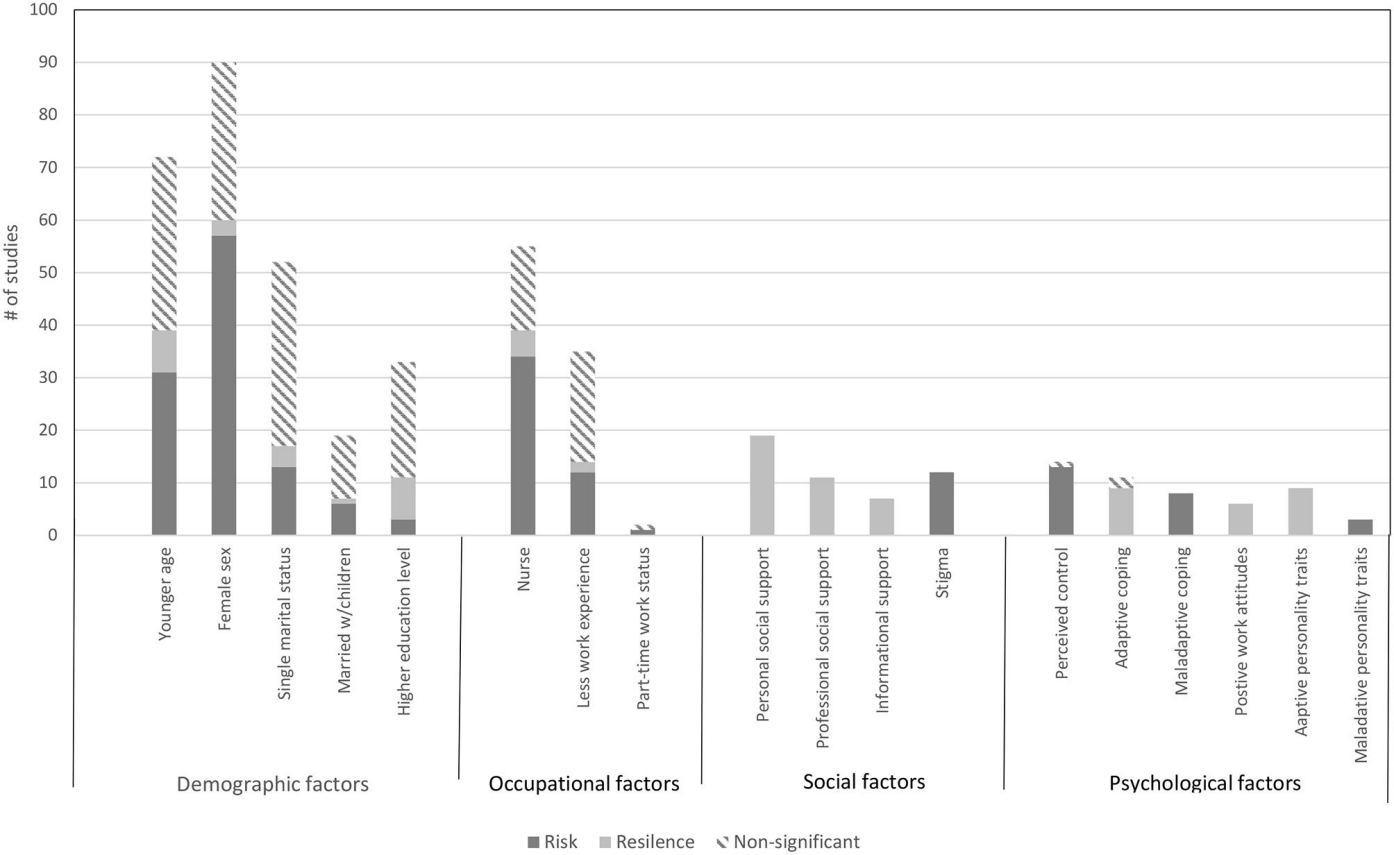
Findings from the studies that examined fixed (demographic and occupational) and modifiable (social and psychological) factors and associations with risk and resilience for psychological distress.

**Figure 3:**
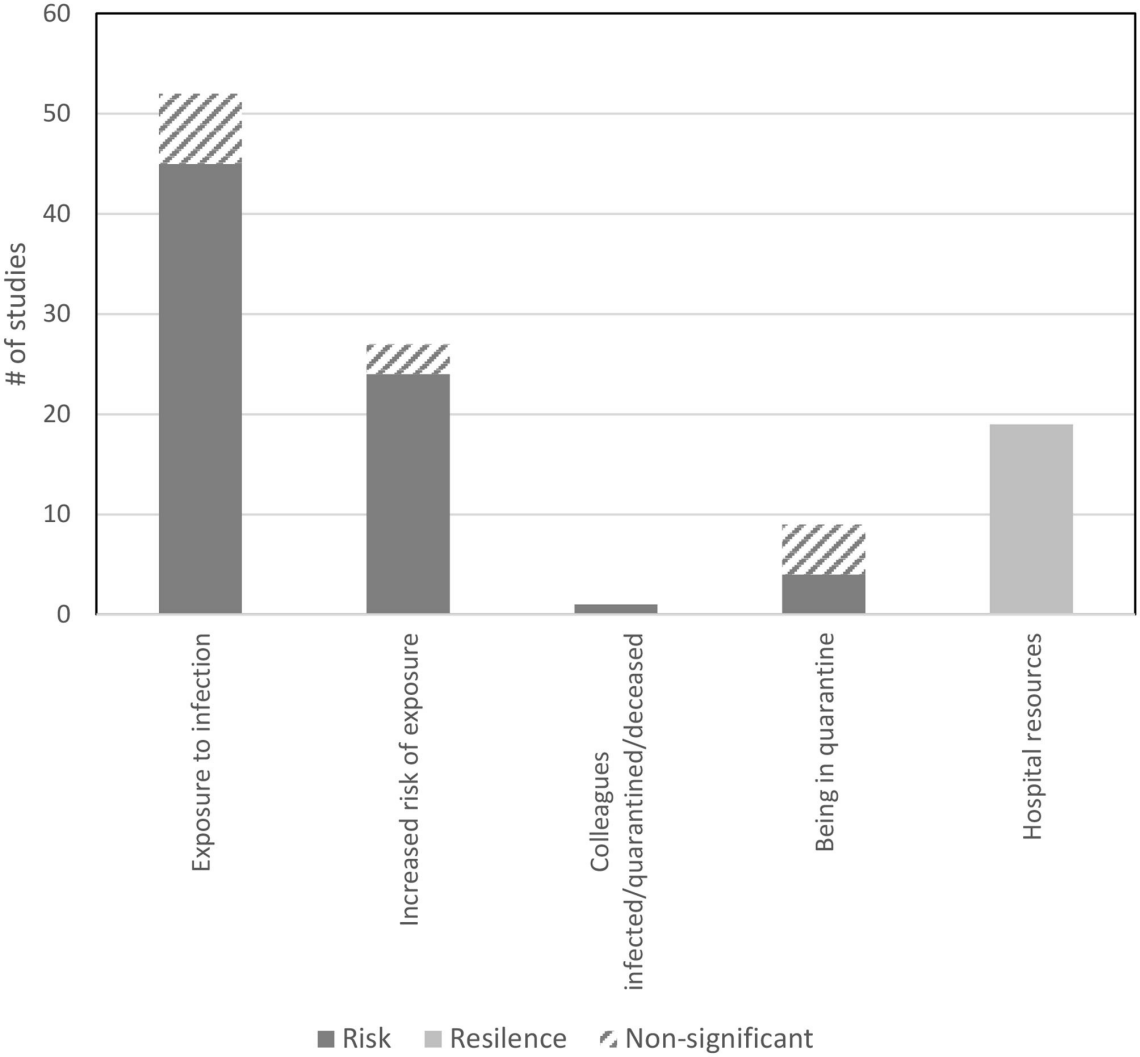
Findings from the studies that examined factors related to infection exposure and associations with risk and resilience for psychological distress.

The search yielded 4621 records, with 138 papers reporting 139 studies (Total *N* = 143,246 HCW) that met inclusion criteria for this Review. Figure 1 presents the complete screening process. Characteristics of the studies are in Table 1. The average sample size was 1,030 (range 82–21,199). The studies included HCW working across 34 countries during COVID-19 (*k* = 107, *N* = 120,711), SARS (*k* = 21, *N* = 18,096), MERS (*k* = 7, *N* = 1,567), H1N1 (*k* = 2, *N* = 2,094), Ebola (*k* = 1, *N* = 143), and H7N9 (*k* = 1, *N* = 102), outbreaks. The rates of psychological distress in HCW varied depending on how distress was measured (Table 1). Figures 2, 3 provide a graphical overview of the weight of the evidence per factor.

**Table 1:** Characteristics of the 139 studies (*N* = 143,246) included in the rapid review.

**Study authors and year**	**Country**	**Study design**	**Sample (% female)**	**Infectious disease**	**Study period**	**Psychological distress measures**	**Rates of distress (%)**	**Risk/resilience factors tested**
Abdulah and Mohammed (23)	Kurdistan	Cross-sectional	209 doctors (25.4)	COVID-19	09/04/2020–14/04/2020	PSS-10 to measure stress	21.1 (Low stress) 69.4 (Moderate stress) 9.6 (high stress)	Age, sex, work experience
Ahmed et al. (24)	China	Cross-sectional	497 nurses (78.87)	COVID-19	18/01/2020–20/01/2020	K-6 to measure non-specific psychological distress	65.0 (moderate to severe psychological distress)	Social support-professional/organizational
Aksoy and Koçak (25)	Turkey	Cross-sectional	758 nurses and midwives (92.70)	COVID-19	1/04/2020–14/04/2020	STAI to measure anxiety	NR	Sex, exposure to confirmed infected cases.
Al Mahyijari et al. (26)	Oman	Cross-sectional	150 doctors and nurses (77.30)	COVID-19	NR	PSS-10 to measure stress, GAD-7 to measure anxiety	30.0 (moderate to severe anxiety)	Sex, age, HCW type
Alan et al. (27)	Turkey	Cross-sectional	416 HCW (79.10)	COVID-19	16/04/2020–20/04/2020	DASS to measure depressive symptoms, anxiety and stress	17.8 (moderate depression) 16.8 (severe depression) 18.1 (extremely severe depression) 17.8 (moderate anxiety) 13.9 (severe anxiety) 22.6 (extremely severe anxiety) 19.7 (moderate stress) 16.6 (severe stress) 7.9 (extremely severe stress	Age, sex, marital status, HCW type, higher education level, direct contact with confirmed infected cases
Arafa et al. (28)	Egypt and Saudi Arabia	Cross-sectional	426 doctors, nurses and HCW-ancillary workers (49.8)	COVID-19	14/04/2020–24/04/ 2020	DASS-21 to measure stress, depressive symptoms and anxiety	69.0 (depression) 59.8 (anxiety) 55.9 (stress)	Sex, age, social support-personal, social support-professional/organizational
Arshad and Islam (29)	Pakistan	Cross-sectional	431 doctors (44.78)	COVID-19	Last week of March 2020	GAD-7 to measure anxiety	27.84 (mild anxiety) 23.90 (moderate anxiety) 9.74 (severe anxiety)	Age, Sex
Azoulay et al. (30)	85 countries (European Society of Intensive Medicine)	Cross-sectional	1,001 HCW (34.20)	COVID-19	30/04/2020–25/5/ 2020	HADS to measure anxiety and depressive symptoms, MBI to measure burnout	46.5 (anxiety) 30.2 (depression)	Age, sex, marital status single vs. married
Babore et al. (31)	Italy	Cross-sectional	595 HCW (80.3)	COVID-19	11/04/2020–16/04/2020	PSS-10 to measure stress	NR	Sex, marital status; married with children, social support-personal, direct contact with infected cases, adaptive and maladaptive coping style, positive work attitudes
Badahdah et al. (32)	Oman	Cross-sectional	509 doctors and nurses (80.30)	COVID-19	1st 2 weeks of April 2020	GAD-7 to measure anxiety, PSS-10 to measure stress	25.9 (moderate to severe anxiety) 56.4 (high stress)	Age, sex, marital status, HCW type, exposure to confirmed infected cases
Barello et al. (33)	Italy	Cross-sectional	376 doctors and nurses (73.70)	COVID-19	5 weeks from the beginning of COVID-19 epidemic in Italy	MBI to measure burnout	37.0 (high emotional exhaustion)	Sex, HCW type
Bates et al. (34)	UK England	Cross-sectional	117 doctors, nurses and allied health professionals (77.00)	COVID-19	3/04/2020–18/04/2020	GAD-7 to measure anxiety, PCL-5 to measure post-traumatic stress disorder	33.0 (anxiety) 17.0 (distress: PTSD)	HCW type
Bettinsoli et al. (35)	Italy	Cross-sectional	580 doctors, nurses and allied health professionals (40.00)	COVID-19	26/03/2020–9/04/2020 Middle of outbreak in Italy	GHQ-12 to measure psychological distress	33.5 (psychological distress)	Sex, HCW type, marital status: married with children, direct contact with infected cases, perceived control, adaptive coping style
Blekas et al. (36)	Greece	Cross-sectional	270 HCW (73.7)	COVID-19	10/04/2020–13/04/2020	PDI to measure levels of distress, PHQ-9 to measure depressive symptoms, PTSD-8 to measure post-traumatic stress disorder	16.7 (distress PTSD)	Age, sex
Bukhari et al. (37)	Saudi Arabia	Cross-sectional	386 HCW (86.00)	MERS	NR	Study specific measure of worry about contracting MERS	33.2 (extremely or very worried)	Sex, direct contact with confirmed infected cases
Cai et al. (38)	China	Cross-sectional	1,521 HCW (75.54)	COVID-19	NR	SCL-90-R to measure psychological distress	14.1 (psychological distress)	Age, sex, marital status: married with children, HCW type, Social support-personal, less work experience, adaptive personality traits
Cai et al. (39)	China	Cross- sectional	534 HCW (68.70)	COVID-19	01/2020–03/2020	Study specific measure of stress	NR	Social support-personal
Caillet et al. (40)	France	Cross-sectional	208 HCW in the ICU (75.00)	COVID-19	8/04/2020–21/04/2020 Peak of the pandemic	HADS to measure anxiety and depressive symptoms, IES-R to measure post-traumatic stress disorder	48.0 (anxiety) 16.0 (depression) 27.0 (distress; PTSD)	Sex, age, HCW type, risk of exposure to confirmed cases
Chan and Huak (41)	Singapore	Cross-sectional	661 doctors and nurses (NR)	SARS	05/2003 2 months after SARS outbreak	IES-R, to measure post-traumatic stress disorder, GHQ-28 to measure distress	27.0 (distress; PTSD)	HCW type, marital status, social support-personal, adequate information, positive work attitude
Chatterjee et al. (42)	India-West Bengal	Cross-sectional	152 doctors (21.70)	COVID-19	28/03/2020–06/04/2020	DASS-21 to measure depressive symptoms, stress and anxiety	34.9 (depression) 39.5 (anxiety) 32.9 (stress)	Age, sex, less work experience, at risk of being in contact with infected patients
Chen et al. (43)	Taiwan	Cross-sectional	128 nurses (100.00)	SARS	During mid-May 2003, at the peak of the SARS outbreak.	IES to measure PTSD, SCL-90-R to measure psychological distress	11.0 (distress: PTSD)	At risk of being in contact with infected patients
Chen et al. (44)	Taiwan	Prospective	116 nurses (98.30)	SARS	May 2003	SAS to measure anxiety, SDS to measure depressive symptoms	NR	Social support-personal, training for dealing with SARS provided
Chen et al. (45)	China	Cross-sectional	902 HCW (68.63)	COVID-19	9/02/2020–11/02/2020 Peak of pandemic	CMBI to measure post-traumatic stress disorder, GAD-7 to measure anxiety, PHQ-9 to measure depressive symptoms	24.5 (moderate-severe anxiety and depression) 16.63 (moderate to severe anxiety) 18.29 (moderate to severe depression)	Sex, HCW type, adaptive and maladaptive coping style, adaptive personality traits
Chen et al. (46)	China and Taiwan	Cross-sectional	12,956 nurses (95.60)	COVID-19	April 2020	MBI GS to measure extent of emotional exhaustion,	24.7 and 23.5 (emotional exhaustion HRW)	Sex, exposure to confirmed infected cases
Chen et al. (47)	China	Cross-sectional	171 HCW (67.83) (94 HRW [74.50], 77 LRW [59.70])	COVID-19	NR	PCL-C to measure post-traumatic stress disorder, GAD-7 to measure anxiety, PHQ-9 to measure depressive symptoms	28.7 (distress; PTSD:HRW) 13.0 (distress; PTSD:LRW) 63.8 (anxiety: HRW) 45.5 (anxiety: LRW) 19.1 (moderate to severe depression: HRW) 6.5 (moderate to severe depression LRW)	Sex, higher education level, HCW type, direct exposure with confirmed infected cases
Chew et al. (48)	Asia-Pacific region	Cross-sectional	1,146 HCW (65.10)	COVID-19	29/04/2020–4/06/2020	DASS-21 to measure stress, depressive symptoms and anxiety, IES to measure post-traumatic stress disorder	NR	Sex
Chong et al. (13)	China	Cross-sectional	1,257 HCW (81.10)	SARS	12/05/2003–27/06/2003 6 weeks during outbreak	IES-R to measure post-traumatic stress disorder, CHQ to measure psychiatric morbidity	75.3 (psychiatric morbidity)	Sex, marital status, HCW type, work experience, exposure to confirmed infected cases
Civantos et al. (49)	Brazil	Cross-sectional	163 doctors (25.80)	COVID-19	14/05/2020–31/05/2020	GAD-7 to measure anxiety, IES-R to measure post-traumatic stress disorder, PHQ-2 to measure depressive symptoms, Mini-Z to measure physician burnout	14.7 (emotional burnout) 19.7 (moderate-severe anxiety) 26.3 (distress; PTSD) 16.3 (depression)	Age, sex
Cunill et al. (50)	Spain	Cross-sectional	1,452 HCW (82.90)	COVID-19	4/04/2020–10/04/2020 Peak of pandemic	GAD-7 to measure anxiety, PHQ-9 to measure depressive symptoms, PHQ-15 to measure physical symptoms related to distress	77.10 (emotional burnout) 63.4 (distress) 88.4 (anxiety) 86.1 (depression)	Sex, HCW type
Demirjian et al. (51)	USA	Cross-sectional	689 doctors (47.00)	COVID-19	3/04/2020–11/04/2020 8 days	Study specific measures for anxiety and stress	61.0 (anxiety)	Sex, Hospital resources/protection/training for the treatment of infection
Di Tella et al. (52)	Italy	Cross-sectional	145 doctors and nurses (72.40)	COVID-19	19/03/2020–05/04/2020	PCL-5 to measure PTSD, BDI-II to measure depressive symptoms, STAI to measure anxiety	NR	Exposure to confirmed infected cases
Dobson et al. (53)	Australia	Cross-sectional	320 HCW (78.50)	COVID-19	16/04/2020–13/05/2020	PHQ-9 to measure depressive symptoms, GAD-7 to measure anxiety, IES-R to measure post-traumatic stress disorder, PFI to measure burnout	2126.2 (distress: PTSD) 31.0 (moderate-severe depression) 71.0 (anxiety) 29.0 (distress: PTSD)	Adaptive personality traits, less work experience, direct contact with confirmed infected cases
Elbay et al. (54)	Turkey	Cross-sectional	442 HCW (56.80)	COVID-19	10/03/2020–15/03/2020	DASS-21 to measure depressive symptoms, stress and anxiety	64.7 (depression) 51.6 (anxiety) 41.2 (stress)	Age, sex, marital status, less work experience, social support-professional/organizational, hospital resources, protection, training, at risk of being in contact with infected patients
Elhadi et al. (55)	Libya	Cross-sectional	745 doctors and nurses (51.90)	COVID-19	18/04/2020–28/04/2020	HADS to measure anxiety and depression	56.3 (depression) 46.7 (anxiety)	Age, sex, marital status, less work experience, stigma
Elkholy et al. (56)	Egypt	Cross-sectional	502 HCW (50.00)	COVID-19	April–May 2020	GAD-7 to measure anxiety, PHQ-9 to measure depressive symptoms, PSS to measure level of perceived stress	76.4 (anxiety) 77.2 (depression) 80.9 (stress)	Sex
Erquicia et al. (57)	Spain	Cross-sectional	395 HCW (73.60)	COVID-19	March–April 2020	DASS-21 to measure stress, depressive symptoms and anxiety, HARS to measure anxiety, MADRS to measure depressive symptoms	31.4 (moderate-severe anxiety) 12.1 (moderate-severe depression) 14.5 (moderate-severe stress)	Age, sex, marital status, direct contact with confirmed infected cases
Fauzi et al. (58)	Malaysia	Cross-sectional	1,050 doctors (71.50)	COVID-19	May 2020 1 month	DASS-21 to measure stress, depressive symptoms and anxiety	31.0 (depression) 29.7 (anxiety) 23.5 (stress)	Perceived control, adaptive coping styles
Fiksenbaum et al. (59)	Canada	Cross-sectional	333 nurses (94.59)	SARS	03/2004–05/2004	Study specific measures on worry about contracting SARS, MBI GS to assess extent of emotional exhaustion	NR	Social support-professional/organizational, direct contact with infected cases, time spent in quarantine
García-Fernández et al. (60)	Spain	Cross-sectional	781 HCW (NR)	COVID-19	29/03/2020–05/04/2020 1 week during the peak of the outbreak	HAM-A to measure anxiety, BDI to measure depressive symptoms, ASDI to measure stress	NR	Work experience, Adequate information, Hospital resources, protection, training
Giardino et al. (61)	Argentina	Cross-sectional	1,059 HCW (72.70)	COVID-19	5/06/2020–25/06/2020	GADS to measure anxiety and depression	81.0 (depression) 76.5 (anxiety)	Age, sex, HCW type, direct contact with confirmed infected cases
Giusti et al. (62)	Italy	Cross-sectional	330 HCW (62.60)	COVID-19	16/04/2020–11/05/2020	STAI to measure anxiety, DASS-21 to measure stress, depressive symptoms and anxiety, IES-6 to measure post-traumatic stress disorder, MBI to measure burnout	71.2 (anxiety) 26.8 (depression) 34.3 (stress) 36.7 (distress; PTSD)	Sex, HCW type, social support-personal, direct contact with confirmed infected cases
Goulia et al. (63)	Greece	Cross-sectional	469 HCW (68.40)	H1N1	1/09/2009–30/09/2009 At the beginning of the second wave of the pandemic	GHQ-28 to measure psychological distress, study specific measure of worry about H1N1	27.5 (mild to severe psychological distress) 56.7 (worry)	HCW type, stigma, adequate information, positive work attitudes
Grace et al. (64)	Canada	Cross-sectional	193 physicians (32.10)	SARS	During the SARS outbreak in 2003	Study specific question about new distressing psychological symptoms	18.1 (new distressing symptoms)	Direct contact with confirmed infected cases
Gupta et al. (65)	India	Cross-sectional	1,124 HCW (36.10)	COVID-19	30/03/2020–2/04/2020 4 days	HADS to measure anxiety and depression	37.2 (anxiety) 31.4 (depression)	Age, sex, marital status, higher education level, HCW type, less experience, direct contact with infected cases, hospital resources, protection, training
Han et al. (66)	China	Cross-sectional	21,199 nurses (98.60)	COVID-19	7/02/2020–10/02/2020	SAS to measure anxiety, SDS to measure depressive symptoms	3.9 (moderate anxiety) 0.8 (severe anxiety) 6.9 (moderate depression) 1.3 (severe depression)	Sex, age, marital status, direct contact with infected cases, at risk of being in contact with infected patients,
Hasan et al. (67)	Pakistan	Cross-sectional	151 doctors (56.30)	COVID-19	30/04/2020–16/05/2020	GAD-7 to measure anxiety	14.6 (moderate anxiety) 3.3. (severe anxiety)	Sex, direct contact with confirmed cases
Ho et al. (68) Sample 1	Hong Kong	Cross-sectional	82 HCW (56.09)	SARS	5/04/03–5/05/03 During height of outbreak	Study specific measures of worry about contracting SARS	NR	Perceived control
Ho et al. (68) Sample 2	Hong Kong	Cross-sectional	97 HCW (82.50)	SARS	Sample 2 08/2003	CIES–R to measure post-traumatic stress disorder	NR	Perceived control
Holton et al. (69)	Australia	Cross-sectional	688 HCW (85.00)	COVID-19	15/05/2020–10/06/2020	DASS-21 to measure stress, depressive symptoms and anxiety	25.0 (psychological distress)	Sex, marital status, less experience, direct contact with confirmed infected cases
Hong et al. (70)	China	Cross-sectional	4,692 nurses (96.90)	COVID-19	8/02/2020–14/02/2020 2 weeks after the authority in Wuhan suspended all public transport on 23/01/2020	PHQ-9 to measure depressive symptoms, GAD-7 to measure anxiety	9.4 (depressive symptoms) 8.1 (anxiety)	Marital status, higher education level, social support-personal, social support-professional and organizational, perceived risk
Hosseinzadeh-Shanjani et al. (71)	Iran	Cross-sectional	200 HCW (80.00)	COVID-19	March 2020–May 2020	DASS-21 to measure stress, depressive symptoms and anxiety	NR	Age, sex, marital status, higher education level,
Hu et al. (72)	China	Cross-sectional	2,014 nurses (87.10)	COVID-19	13/02/2020-24/02/2020 At the peak of the outbreak	MBI-HSS to measure burnout, SAS to measure anxiety, SDS to measure depressive symptoms	60.5 (emotional exhaustion) 14.3 (anxiety) 10.7 (depression)	Age, sex, marital status, social support-personal, higher education level, less work experience, social support-personal, perceived control, adaptive personality traits, at risk of being in contact with infected patients, hospital resources, protection, training
Huang et al. (73)	China	Cross-sectional	587 mixture of radiology staff (52.00)	COVID-19	7/02/2020–9/02/2020	CPSS to measure stress, CSAS to measure anxiety	NR	Sex, marital status
Huffman et al. (74)	USA	Cross-sectional	720 HCW (NR)	COVID-19	21/04/2020 for 3 weeks Survey was open during the state of Indiana's peak day of COVID-19 cases on 26/04/2020	Grit-S to measure perceived grit	NR	Adaptive coping style, hospital resources, protection, training
Jain et al. (75)	India	Cross-sectional	512 anaesthesiologists (44.30)	COVID-19	12/05/2020–22/05/2020	GAD-7 to measure anxiety	74.2 (anxiety)	Age, sex, marital status, less work experience, direct contact with infected cases, hospital protection (PPE) for treatment of infected cases
Ji et al. (76)	Sierra Leone	Cross-sectional	143 medical staff and students (49.50)	Ebola (EVD)	13/02/2015–19/03/2015 During Ebola outbreak	SCL-90-R to measure psychological symptoms	NR	Educational level
Jo et al. (77)	South Korea	Cross-sectional	253 HCW (83.00)	COVID-19	NR	IES-R to measure post-traumatic stress disorder	NR	Sex, HCW type
Juan et al. (78)	China	Cross-sectional	456 doctors and Nurses (70.60)	COVID-19	01/02/2020–14/02/2020	IES-R to measure post-traumatic stress disorder, GAD-7 to measure anxiety, PHQ-9 to measure depressive symptoms	37.5 (psychological distress) 31.6 (anxiety) 29.6 (depression)	Sex, age, level of education, HCW type, direct contact with infected cases, risk of contact with infected cases, stigma, social support-personal, time spent in quarantine
Jung et al. (79)	South Korea	Cross-sectional	147 nurses (NR)	MERS	1/10/2015–30/11/2015 Shortly after the MERS epidemic ended	IES-RK to measure post-traumatic stress disorder, GHQ-12 to measure mental health, study specific measure of stress	57.1 (distress: PTSD)	Social support-Professional/organizational
Khattak et al. (80)	Pakistan	Cross-sectional	380 nurses (84.21)	COVID-19	NR	CAPS to measure post-traumatic stress disorder,	NR	Social support-organizational/ professional
Kim and Choi (81)	South Korea	Cross-sectional	223 ED nurses (93.50)	MERS	20/07/2015–31/07/2015. 2 months after the outbreak of MERS during uncontrolled disease period	OLBI to assess MERS-related burnout	NR	Age, sex, marital status, level of education, work experience, direct contact with infected cases, social support-personal, hospital resources, protection, training
Kim et al. (82)	South Korea	Cross-sectional	112 nurses (88.30)	MERS	30/06/2015–10/07/2015	IES to measure post-traumatic stress disorder, MBI-HSS to measure burnout.	50.0 (distress: PTSD)	Age, sex, marital status, higher level of education, less work experience
Koh et al. (83)	Singapore	Cross-sectional	7,614 HCW (82.00)	SARS	05/2003–07/2003 Toward the tail end of the pandemic	IES to measure post-traumatic stress disorder; single item to measure perceived stress at work	56.0 (stress)	HCW type, marital status, Stigma, exposure to SARS
Lai et al. (84)	China	Cross-sectional	1,257 HCW (76.70)	COVID-19	29/01/20–3/02/20 During pandemic	PHQ-9 to measure depression, GAD-7 to measure anxiety, CIES-R to measure post-traumatic stress disorder	50.4 (depression) 44.6 (anxiety) 71.5 (distress: PTSD)	Sex, HCW type, direct contact with confirmed infected cases
Lee et al. (85)	South Korea	Cross-sectional	359 HCW (81.90)	MERS	05/32015–12/2015 During the outbreak	IES-R to measure post-traumatic stress	51.0 (distress: PTSD)	Sex, age, HCW type, at risk of being in contact with infected patients, time spent in quarantine
Leng et al. (86)	China	Cross-sectional	90 nurses (72.20)	COVID-19	11/03/2020–18/03/2020 At the time of the survey, nurses had worked in Wuhan for at least 32 days	CPSS to measure psychological distress, PCL-C to measure post-traumatic stress disorder	5.6 (distress: PTSD)	Sex, age, marital status, level of education, less work experience
Li et al. (87)	China	Cross-sectional	908 HCW (75.55)	COVID-19	3/02/2020-24/02/2020 Survey began 10 days after state of emergency declared on 23/01/2020	SAS to measure anxiety, SDS to measure depressive symptoms	24.34 (anxiety) 32.93 (depression)	Less work experience, direct contact with confirmed infected cases
Li et al. (88)	China	Cross-sectional	225 reserve medics (72.0)	COVID-19	4/04/2020–6/04/2020	IES-R to measure post-traumatic stress disorder, DASS-21 to measure depressive symptoms, stress and anxiety	46.7 (depression) 35.6 (anxiety) 16.0 (stress) 31.6 (distress: PTSD)	Sex, age social support-professional/ organizational
Li et al. (89)	China	Cross-sectional	356 nurses (86.2)	COVID-19	01/2020–03/2020	PSS-10 to measure stress, PCL-5 to measure post-traumatic stress disorder	NR	Age, marital status, level of education, les work experience, job role, direct contact with infected cases, adaptive personality traits
Liao et al. (90)	China	Cross-sectional	1,092 nurses (99.51)	COVID-19	02/2020	SSAR to measure stress	NR	Age, sex, marital status, level of education, social support-personal, perceived control
Lin et al. (91)	China	Cross-sectional	114 HCW (79.80)	COVID-19	02/2020	HADS to measure anxiety and depression	NR	Adaptive and maladaptive coping styles, adaptive personality traits
Liu et al. (92)	China	Cross-sectional	549 HCW (75.2)	SARS	In 2006, 3 years after Beijing's SARS outbreak	CES-D to measure depressive symptoms	22.8 (moderate or severe depression)	Sex, age, marital status, altruistic perspective toward work, exposure to infection, being quarantined
Liu et al. (93)	China	Cross-sectional	512 HCW (79.96)	COVID-19	10/02/20–20/02/20 During pandemic	SAS to measure anxiety	12.5 (mild to severe anxiety)	Sex, age, marital status, level of education, HCW type, direct contact with confirmed infected cases
Liu et al. (94)	China	Cross-sectional	1,090 HCW (80.20)	COVID-19	24/02/2020–9/03/2020	PSS-10 to measure stress, GAD-7 to measure anxiety, PHQ-9 to measure depressive symptoms	13.3 (anxiety) 18.4 (depression) 23.9 (anxiety and depression)	Age, sex, marital status, HCW type, level of education, less experience, social support-personal
Liu et al. (95)	China	Cross-sectional	2,031 doctors and Nurses (85.52)	COVID-19	17/02-2020–23/02/2020	DASS-21 to measure stress, depressive symptoms and anxiety	14.81 (depression) 18.3 (anxiety) 9.98 (stress)	Sex, age, HCW type, role, level of education, direct contact with confirmed infected cases
Lu et al. (96)	Taiwan	Cross-sectional	127 HCW (58.27)	SARS	07/2003–03/2004	CHQ to assess psychiatric morbidity	17.3 (psychiatric morbidity)	Neuroticism
Lu et al. (97)	China	Cross-sectional	2,042 HCW (77.90)	COVID-19	25/02/2020–26/02/2020	HAM-A to measure anxiety, HAM-D to measure depressive symptoms	NR	Direct contact with confirmed infected cases
Magnavita et al. (98)	Italy	Cross-sectional	595 HCW (70.10)	COVID-19	27/03/2020–30/04/2020	GADS to measure anxiety and depression	16.6 (anxiety) 20.3 (depression)	Age, sex, exposure to confirmed infected cases
Maraqa et al. (99)	Palestine	Cross-sectional	430 doctors, nurses, and allied health professionals (54.80)	COVID-19	29/03/2020–15/04/2020	Study specific measure of stress	74.0 (stress)	Age, sex, HCW type, marital status; married with children, direct contact with infected cases, social support-organizational, hospital resources, protection, training
Martínez-López et al. (100)	Spain	Cross-sectional	157 HCW (79.00)	COVID-19	6/04/2020–19/04/2020 Middle of lockdown in Spain and at peak of pandemic	MBI to measure burnout		Age, sex, HCW type, hospital resources (PPE) for treatment of infection
Marton et al. (101)	Italy	Cross-sectional	458 HCW (NR)	COVID-19	24/03/2020–13/05/2020 Phase 1 of Italian COVID-19 emergency	GHQ-12 to measure psychological distress	21.26 (psychological distress)	Age, less experience, perceived control
Master et al. (102)	China	Cross-sectional	263 nurses (76.70)	COVID-19	3/02/2020–11/02/2020	GHQ-12 to measure psychological distress, IES-R to measure post-traumatic stress disorder	25.1 (psychological distress)	Sex, age, level of education, marital status, less experience, adaptive and maladaptive coping styles, stigma, social support-personal, hospital resources, protection, training
Matsuishi et al. (103)	Japan	Cross-sectional	1,625 HCW (75.60)	H1N1	16/03/2009–31/07/2009 Approximately 1 month after the peak of outbreak	IES to measure post-traumatic stress disorder, study specific measures on stress	NR	Age, sex, HCW type, at risk of being in contact with infected patients
Maunder et al. (104)	Canada	Cross-sectional	1,557 HCW (74.60)	SARS	12/05/2003–20/06/2003 During the outbreak	IES to measure psychological stress	NR	Direct contact with infected cases, stigma
Maunder et al. (14)	Canada	Cross-sectional	587 HCW (87.80)	SARS	23/10/2004–30/09/2005 13–26 months after outbreak	IES to measure post-traumatic stress disorder, K10 to measure non-specific psychological distress, MBI-EE to measure burnout	NR	Work experience, stigma, maladaptive coping styles, maladaptive personality traits, direct contact with infected cases, time spent in quarantine
McAlonan et al. (16)	Hong Kong	Cross-sectional across 2 time points	T1 = 176 T2 = 184 HCW (73.25, T1; 64.50, T2)	SARS	T1: 15/04/2003–15/05/2003. During the peak period of hospital admissions for SARS. T2: 2004	PSS-10 to measure stress, DASS-21 to measure stress, depressive symptoms and anxiety, IES-R to measure post-traumatic stress disorder	NR	At risk of being in contact with infected patients
Mo et al. (105)	China	Cross-sectional	200 nurses (89.00)	COVID-19	22/02/2020	SAS to measure subjective anxiety, SOS to measure stress	NR	Sex, marital status, level of education, perceived control, direct contact with confirmed infected cases
Mosheva et al. (106)	Israel	Cross-sectional	1,106 doctors (49.0)	COVID-19	19/03/2020–22/03/2020 Whilst confirmed cases were rising	Study specific measures of stress	NR	Marital status, hospital training for treatment of infection, adaptive personality traits
Nickell et al. (107)	Canada	Cross-sectional	510 HCW (78.80)	SARS	10/04/2003–22/04/2003 Conducted during the peak of the initial phase of the SARS outbreak	GHQ-12 to measure psychological distress	29.0 (distress)	HCW type, part-time work status
Park et al. (108)	South Korea	Cross-sectional	187 nurses (100.00)	MERS	30/08/2015–21/09/2015 Conducted during MERS epidemic	PSS to measure level of perceived stress, SF-36 MH to measure mental health status	NR	Marital status, work experience, stigma, adaptive personality traits
Park et al. (109)	South Korea	Cross-sectional	1,003 HCW (77.10)	COVID-19	2/04/2020–10/04/2020 Whilst cases were increasing	PHQ-9 to measure depressive symptoms, GAD-7 to measure anxiety	NR	HCW type, stigma, direct contact with infected cases, time spent in quarantine
Phua et al. (110)	Singapore	Cross-sectional	96 doctors and nurses (64.60)	SARS	1/11/2003–14/11/2003 6 months after the end of the outbreak	IES to measure post-traumatic stress disorder, GHQ-28 to measure psychiatric morbidity	18.8 (psychiatric morbidity), 17.7 (distress: PTSD)	HCW type, maladaptive coping styles
Podder et al. (111)	India	Cross-sectional	384 doctors (44.53)	COVID-19	03/04/2020–10/04/2020	PSS-10 to measure stress	85.6 (moderate and high stress)	Age, sex, marital status
Poon et al. (112)	Hong Kong	Cross-sectional	1,926 HCW (NR)	SARS	05/2003–06/2003 Diagnosis of the first case of SARS occurred on 12/03/2003. Hong Kong declared SARS-free on 23/06/2003	STAI to measure anxiety, MBI-EE to measure emotional burnout	NR	HCW type, contact with confirmed infected cases
Pouralizadeh et al. (113)	Iran	Cross-sectional	441 nurses (95.20)	COVID-19	7/04/2020–12/04/2020	GAD-7 to measure anxiety, PHQ-9 to measure depressive symptoms	38.8 (anxiety) 37.4 (depression)	Age, sex, marital status, level of education, less work experience, risk of contact with infected cases, hospital resources, protection, training
Prasad et al. (114)	USA	Cross-sectional	347 HCW (90.80)	COVID-19	14/04.20202–25/04/2020	GAD-7 to measure anxiety, Mini Z to measure burnout, IES to measure distress, PHQ-2 to measure depressive symptoms	69.5 (anxiety) 84.1 (mild distress) 22.8 (depression)	Age, HCW role
Que et al. (115)	China	Cross-sectional	2,285 HCW (69.06)	COVID-19	16/02/2020–23/02/2020 Early stage of COVID-19 pandemic	GAD-7 to measure anxiety, PHQ-9 to measure depressive symptoms	46.0 (anxiety) 44.4 (depression)	Sex, at risk of being in contact with infected patients
Rodriguez-Menéndez et al. (116)	Spain	Cross-sectional	1,407 HCW (71.50)	COVID-19	11/05/2020–31/05/2020	GHQ-28 to measure distress, SASR to measure perceived anxiety	24.7 (acute stress)	Sex, age, HCW type, Hospital resources, protection, training, Social support – professional/organizational, adequate information
Romero et al. (117)	Spain	Cross-sectional	3,109 HCW (NR)	COVID-19	09/04/2020–19/04/2020 10 days during the outbreak	Study specific measure of stress	NR	Age
Rossi et al. (118)	Italy	Cross-sectional	1,379 HCW (77.20)	COVID-19	27/03/2020–31/03/2020 Days immediately preceding the peak 77.2 of the COVID-19 outbreak in Italy	GAD-7 to measure anxiety, PSS to assess perceived stress, PHQ-9 to measure depressive symptoms, GPS to assess post-traumatic stress symptoms (PTSS)	49.4 (distress: PTSD)	Sex, age, HCW type, colleagues being infected, quarantined, deceased
Ruiz-Fernández et al. (119)	Spain	Cross-sectional	506 doctors and nurses (76.70)	COVID-19	30/03/2020–16/04/2020	PSS-14 to measure stress	NR	Sex, marital status, HCW type, part-time work, direct contact with confirmed infected cases
Sagaon-Teyssier et al. (120)	Mali	Cross-sectional	135 HCW (39.30)	COVID-19	6/04/2020–11/04/2020	GAD-7 to measure anxiety, PHQ-9 to measure depressive symptoms	NR	Sex, marital status, HCW type, hospital resources, protection, training
Sahin et al. (121)	Turkey	Cross-sectional	939 HCW (66.00)	COVID-19	23/04/2020–23/05/2020	GAD-7 to measure anxiety, IES-R to measure post-traumatic stress disorder, PHQ-9 to measure depressive symptoms	77.6 (depression) 60.2 (anxiety) 76.4 (psychological distress)	Sex, age, HCW type, less work experience, risk of contact with infected cases
Saricam (122)	Turkey	Cross-sectional	123 nurses (74.00)	COVID-19	10/04/2020–20/04/2020	STAI to measure anxiety	46.3 (anxiety)	Sex, age, marital status, less work experience, direct contact with confirmed infected cases
Shahrour and Dardas (123)	Jordan	Cross-sectional	448 nurses (73.00)	COVID-19	NR	SAS to measure anxiety, BSI-18 to measure psychological distress	64.0 (acute stress) 41.0 (significant psychological distress)	Sex, age, perceived control
Shechter et al. (124)	USA	Cross-sectional	657 HCW (70.90)	COVID-19	09/04/2020–24/04/2020	GAD-2 to measure anxiety, PHQ-2 to measure depressive symptoms, PC-PTSD to measure acute stress	57.0 (acute stress) 48.0 (depression) 33.0 (anxiety)	HCW type
Si et al. (125)	China	Cross-sectional	863 HCW (70.70)	COVID-19	23/02/2020–5/03/2020	IES-6 to measure post-traumatic stress disorder, DASS-21 to measure stress, depressive symptoms and anxiety	13.6 (depression) 13.9 (anxiety) 8.6 (stress)	Sex, age, marital status, level of education, HCW type, direct contact with infected cases, time spent in quarantine
Son et al. (126)	South Korea	Cross-sectional	153 HCW hospital staff (74.30)	MERS	25/08/2015–14/09/2015 Approximately 1 month after the end of the outbreak on 28/07/2015	IES-RK to measure post-traumatic stress disorder	18.6 (distress: PTSD)	Loss of control and perceived risk, adaptive coping styles and ability
Song et al. (127)	China	Cross-sectional	14,825 doctors and nurses (64.30)	COVID-19	28/02/2020–18/03/2020	CES-D to measure depression, PCL-5 to measure post-traumatic stress disorder (PTSD)	25.2 (depression) 9.1 (PTSD)	Age, sex, marital status, HCW type, less work experience, social support-personal
Sorokin et al. (128)	Russia	Cross-sectional	1,800 HCW Phase 1: 223 (79.50) Phase 2: 1577 (89.50)	COVID-19	1st week of self-isolation Phase 1: 30/03/2020–5/04/2020 Phase 2: 4/05/2020–10/05/2020	PSM-25 to measure anxiety and distress	NR	Marital status, HCW type, direct contact with confirmed infected cases
Stojanov et al. (129)	Serbia	Cross-sectional	201 HCW Group 1: 118 (65.60) Group 2: 83 (66.30)	COVID-19	NR	GAD-7 to measure anxiety, SDS to measure depressive symptoms	NR	Direct contact with confirmed infected cases
Styra et al. (130)	Canada	Cross-sectional	248 HCW (87.02)	SARS	16/06/2003–9/07/2003	IES-R to measure post-traumatic stress disorder	NR	Age, sex, marital status, work experience, adequate information, at risk of being in contact with infected patients
Sun et al. (131)	China	Cross-sectional	536 HCW (69.00)	COVID-19	2/03/2020–6/03/2020	PHQ-9 to measure depressive symptoms, GAD-7 to measure anxiety,	NR	Age, sex, marital status, colleagues being infected/quarantined, direct contact with confirmed infected cases
Sun et al. (132)	China	Cross-sectional	442 HCW (84.30)	COVID-19	31/01/2020–4/02/2020	IES to measure post-traumatic stress disorder	NR	Age, sex, marital status, HCW type, less work experience, at risk of being in contact with infected patients, direct contact with infected cases, time spent in quarantine
Surrati et al. (133)	Saudi Arabia	Cross-sectional	122 HCW (64.40)	COVID-19	04/2020–05/2020	HADS to measure anxiety and depression, PSS to measure perceived stress	35.6 (anxiety) 27.9 (depression) 72.8 (moderate stress)	Sex, HCW type, direct contact with infected cases, hospital resources, protection, training
Tam et al. (134)	Hong Kong	Cross-sectional	652 front-line Hospital HCW (79.00)	SARS	06/2003–08/2003	GHQ-12 to measure psychological distress, Study specific measure for job-related stress	56.7 (psychological distress) 68.0 (stress)	HCW type, age, sex, social support-personal, direct contact with infected cases, hospital resources, protection, training
Tan et al. (135)	Singapore	Cross-sectional	3,075 HCW (71.50)	COVID-19	29/05/2020–24/06/2020	OLBI to measure burnout, HADS to measure anxiety and depression	NR	Sex, HCW type, level of education, positive work attitudes
Tang et al. (136)	China	Cross-sectional	102 HCW (66.70)	H7N9	01/2015 and 05/2016	PCL-C to measure post-traumatic stress disorder	20.6 (distress: PTSD)	Age, sex, HCW type, direct contact with infected cases, hospital resources, protection, training
Teshome et al. (137)	South Ethiopia	Cross-sectional	798 HCW (39.60)	COVID-19	20/05/2020–20/06/2020	GAD-7 to measure anxiety	NR	Direct contact with confirmed infected cases
Teskin et al. (138)	Turkey	Cross-sectional	452 HCW (66.20)	COVID-19	20/05/2020–10/06/2020	HADS to measure anxiety and depression	NR	Stigma
Tselebis et al. (139)	Greece	Cross-sectional	150 nurses (80.00)	COVID-19	5/2020 Last 2 weeks	PSS to measure perceived stress	50.3 (stress)	Age, sex, less experience, social support-personal, direct contact with confirmed infected cases
Tu et al. (140)	China	Cross-sectional	100 nurses (100.00)	COVID-19	07/02/2020–25/02/2020 In the initial stage of the outbreak when there was a shortage of nurses	GAD-7 to measure anxiety, PHQ-9 to measure depressive symptoms	40.0 (anxiety) 46.0 (depression)	Age, marital status, level of education, less work experience
Uyaroglu et al. (141)	Turkey	Cross-sectional	113 doctors (46.90)	COVID-19	1/04/2020–14/04/2020	GAD-7 to measure anxiety, Beck Inventory to measure anxiety and depressive symptoms	NR	Sex, age, marital status, direct contact with confirmed infected cases
Vagni et al. (142)	Italy	Cross-sectional	210 HCW (57.10)	COVID-19	NR	STSS to measure work related stress, study specific measure (Emergency Stress Questionnaire) of stress	NR	Age, sex, HCW type, adaptive coping styles, adequate information
Veeraraghavan and Srinivasan (143)	India	Cross-sectional	100 doctors (44.00)	COVID-19	04/2020–05/2020 Before the peak of the pandemic	Beck Depression Inventory to measure anxiety and depression,	14.0 (moderate anxiety) 15.0 (moderate depression) 2.0 (severe depression)	Sex, direct contact with confirmed infected cases
Verma et al. (144)	Singapore	Cross-sectional	721 doctors (38.80)	SARS	05/2003 2 months after the first case of SARS was reported in Singapore	GHQ-28 to measure psychological distress, IES-R to measure post-traumatic stress disorder	14.1 (psychological distress)	Age, stigma, direct contact with confirmed infected cases
Wang et al. (145)	China	Cross-sectional	202 nurses (87.60)	COVID-19	02/2020–03/2020	PCL-C to measure PTSD	16.8 (distress: PTSD)	Sex, marital status, level of education, adaptive coping styles and adaptability, maladaptive coping styles, positive work attitudes
Wang et al. (146)	China	Cross-sectional	1,045 HCW (85.80)	COVID-19	02/02/2020–03/02/2020	HADS to measure anxiety and depression, PSS-14 to measure perceived stress	13.6 (moderate to severe depression) 20.0 (moderate to severe anxiety)	Sex, HCW type, level of education, less experience, direct contact with infected cases, risk of being in contact with infected cases
Wilson et al. (147)	India	Cross-sectional	350 HCW (46.60)	COVID-19	10/04/2020–25/04/2020	GAD-7 to measure anxiety, PHQ-9 to measure depressive symptoms, PSS-10 to measure distress	17.7 (moderate and severe anxiety) 11.4 (severe depression) 3.7 (high levels of stress)	Sex
Wong et al. (148)	Hong Kong	Cross-sectional	466 ED nurses and doctors (65.70)	SARS	24/06/2003–24/07/2003	Study specific measures on distress caused by SARS	NR	HCW type, loss of control and perceived risk
Xiao et al. (10)	China	Cross-sectional	180 HCW treating patients with COVID-19 (71.70)	COVID-19	01/2020–02/2020	SASR to measure perceived stress, SAS to measure anxiety	NR	Social support-personal, perceived control
Xing et al. (149)	China	Cross-sectional	309 HCW (97.40)	COVID-19	7/02/2020–21/02/2020	SAS to measure anxiety, SDS to measure depression,	28.5 (anxiety) 56.0 (depression)	Age, marital status, level of education, HCW type, direct contact with confirmed infected cases
Xiong et al. (150)	China	Cross-sectional	223 Nurses (97.30)	COVID-19	16/02/2020–25/02/2020	GAD-7 to measure anxiety, PHQ-9 to measure depressive symptoms	40.8 (anxiety) 26.4 (depression)	Age, sex, level of education, less work experience, role type, direct contact with infected cases„ perceived control
Yao et al. (151)	China	Cross-sectional	1,002 HCW (85.20)	COVID-19	1/02/2020–20/02/2020	GHQ-12 to measure psychological distress	61.1 (psychological distress)	Age, sex, marital status, level of education, HCW type, less work experience, direct contact with infected cases, risk of contact with infected cases
Yin et al. (152)	China	Cross-sectional	377 HCW (61.50)	COVID-19	01/02/2020–05/02/2020 During the early stages of the pandemic	PCL-5 to measure post-traumatic stress symptoms (PTSS)	3.8 (distress: PTSD)	Sex, education level, HCW type, direct contact with confirmed infected cases
Yörük and Güler (153)	Turkey	Cross-sectional	377 midwives and nurses (NR)	COVID-19	30/05/2020–13/06/2020 2 weeks	MBI-HSS to measure burnout, Beck Depression Inventory to measure depression, PSS to measure perceived stress	31.8 (depression)	Age, level of education, marital status, less work experience, direct contact with infected cases, adaptive personality traits
Youssef et al. (154)	Egypt	Cross-sectional	540 HCW (45.60)	COVID-19	04/2020	DASS-21 to measure depressive symptoms, stress and anxiety	37.2 (mild-severe stress) 59.0 (depression) 42.6 (anxiety)	Age, sex, marital status, level of education, less work experience
Zhang et al. (155)	China	Cross-sectional	927 HCW (64.96)	COVID-19	19/02/2020–06/03/2020 8 weeks after the outbreak in Wuhan	SCL-90-R to measure psychological symptoms, PHQ-4 to measure anxiety and depressive symptoms	NR	Sex, at risk of being in contact with infected patients
Zhang et al. (156)	China	Cross-sectional	678 HCW (85.05)	COVID-19	6/06/2020–13/06/2020	PCL-C to measure post-traumatic stress disorder, HADS to measure depression and anxiety	41.87 (anxiety) 27.61 (depression)	Sex, age, marital status, level of education, HCW type, direct contact with confirmed infected cases, time spent in quarantine, social support-personal

*COVID-19, Severe Acute Respiratory Syndrome Coronavirus-2 (SARS-CoV-2); H1N1, influenza A virus subtype H1N1; MERS, Middle East respiratory syndrome; SARS, severe acute respiratory syndrome; ED, Emergency Department; HCW, A mixture of nurses, doctors, and health related staff in a hospital; NR, not reported; HRW, high-risk workers in COVID wards; LRW, low-risk workers in non-COVID wards*.

*Measures used in studies: ASDI, Acute Stress Disorder Inventory; BDI, Beck Depression Inventory; BDI-II, Beck Depression Inventory 1996 revision; BSI-18, Brief Symptom Inventory; CAPS, Clinician Administered PTSD Scale; CES-D, Center for Epidemiology Scale for Depression; CHQ, Chinese Health Questionnaire; CMBI, Chinese version of Maslach Burnout Inventory; CIES-R, Chinese Impact of Event Scale—Revised; CPSS, Chinese Perceived Stress Scale; CSAS, Chinese Self-Rating Anxiety Scale; DASS, Depression Anxiety and Stress Scale 42-item; DASS-21, Depression; Anxiety and Stress Scale 21-item; GAD-2, Generalized Anxiety Disorder Scale 2-item; GAD-7, Generalized Anxiety Disorder scale 7-item; GADS, Goldberg Anxiety and Depression Scale; GHQ-12, General Health Questionnaire 12-item; GHQ-28, General Health Questionnaire 28-item; GPS, Global Psychotrauma Screen; Grit-S, Short Grit Scale; HADS, Hospital Anxiety and Depression Scale; HAM-A:HAM-A; Hamilton Anxiety Scale; HAM-D, Hamilton Depression Scale; HARS, Hamilton Rating Scale for Anxiety; IES, Impact of Events Scale; IES-6, Impact of Event Scale for Post-Traumatic Stress Disorder 6-item; IES-R, Impact of Events Scale for Post-Traumatic Stress Disorder; IES-RK, Impact of Event Scale revised Korean version; K-10, Kessler Psychological Distress Scale 10-item; K-6, Kessler Psychological Distress Scale 6-item; MADRS, Montgomery-Asberg Depression Rating Scale; MBI, Maslach Burnout Inventory; MBI-EE, emotional exhaustion scale of the Maslach Burnout Inventory; MBI GS, Maslach Burnout Inventory—General Survey; MBI HSS, Maslach Burnout Inventory-Human Services Survey; Mini-Z, Z Clinician Questionnaire (for “Zero” Burnout); OLBI, Oldenburg Burnout Inventory; PCL-5; Post-traumatic Stress Disorder Check List (for DSM 5); PCL-C, Post-Traumatic Stress Disorder Checklist-Civilian Version; PDI, Peritraumatic Distress Inventory; PHQ-2, Patient Health Questionnaire 2-item; PHQ-4, Patient Health Questionnaire 4-item; PHQ-9, 9-item Patient Health Questionnaire; PHQ-15, Patient Health Questionnaire Physical Symptoms 15-item; PC-PTSD, Primary Care Post-Traumatic Stress Disorder Screen for DSMIV; PSS, Perceived Stress Scale; PSS-10, Perceived Stress Scale 10-item; PSS-14, Perceived Stress Scale 14-item; Psychological Stress Measure 25-item; PTSD-8, Post-Traumatic Stress Disorder 8-item; SAS, Self-Rating Anxiety Scale; SASR, Stanford Acute Stress Reaction scale; SCL-90-R, Symptoms Checklist 90-items; Revised; Chinese version; SDS, Zung Self-Rating Depression Scale Chinese version; SF-36 MH, Short Form Survey mental health component; SOS, Stress Overload Scale; STAI, State-Trait Anxiety Inventory; STSS, Secondary Traumatic Stress Scale*.

### Methodological Quality

The quality of the studies ranged from moderate to high, with no studies rated as having low quality. The majority of the 139 studies were rated as having high quality (118; 84.9%), and 21 studies were rated as having a moderate quality (15.1%). Inter-rater agreement was high, 90.65% agreement, Cohen's Kappa = 0.642 (see Supplementary Table 2).

### Sociodemographic Factors

Seventy-two studies examined age as a predictor of psychological distress among HCW during an epidemic (see Table 2). Of these, 39 found that age was a significant risk factor for distress. In two studies of HCW during the SARS outbreak, staff who were younger than 33 experienced greater stress, but not greater psychiatric morbidity, compared to older staff (134), and staff under 35 were more likely to report severe depressive symptoms 3 years after the outbreak (92). In another study, medical staff who were between 20 and 30 years old and exposed to patients with H7N9 had elevated post-traumatic stress disorder scores compared to older staff (157). Similarly, general practitioners in working during the SARS outbreak who met psychiatric caseness for PTSD were more likely to be younger (144). In a study during the H1N1 outbreak, hospital staff who were in their 20's had greater anxiety about becoming infected than did older staff (103). During COVID-19, HCW who were younger were more likely to experience higher levels of post-traumatic stress symptoms, depression, anxiety, and acute stress compared to older HCW (23, 26–28, 30, 32, 42, 49, 54, 55, 57, 61, 65, 71, 75, 78, 89, 90, 117, 118, 121, 123, 127, 131, 149, 153, 154). In contrast, eight studies conducted during COVID found that HCW who were older were at greater risk of experiencing higher levels of psychological distress (40, 66, 86, 95, 102, 114, 122, 132). Lastly, 33 studies found that age was not a significant predictor of distress in HCW during the SARS, MERS or during the COVID-19 outbreaks (Table 2).

**Table 2:** Overview of the evidence for the factors associated with risk and resilience for psychological distress in health-care workers.

**Factor**	**Evidence for risk**	**Evidence for resilience**	**Non-significant findings**
**Fixed—Demographics**			
Younger age	Abdulah and Mohammed (23), Al Mahyijari et al. (26), Alan et al. (27), Arafa et al. (28), Azoulay et al. (30), Badahdah et al. (32), Chatterjee et al. (42), Civantos et al. (49), Elbay et al. (54), Elhadi et al. (55), Erquicia et al. (57), Giardino et al. (61), Gupta et al. (65), Hosseinzadeh-Shanjani et al. (71), Jain et al. (75), Juan et al. (78), Li et al. (89), Liao et al. (90), Liu et al. (92), Matsuishi et al. (103), Romero et al. (117), Rossi et al. (118), Sahin et al. (121), Shahrour and Dardas (123), Song et al. (127), Sun et al. (131), Tam et al. (134), Tang et al. (157), Verma et al. (144), Xing et al. (149), Yörük and Güler (153), Youssef et al. (154)	Caillet et al. (40), Han et al. (66), Leng et al. (86), Liu et al. (95), Master et al. (102), Prasad et al. (114), Saricam (122), Sun et al. (132)	Arshad et al. (29), Blekas et al. (36), Cai et al. (38), Chen et al. (47), Chen et al. (45), Chew et al. (48), Chong et al. (13), Dobson et al. (53), Elkholy et al. (56), Hu et al. (72), Kim and Choi (81), Kim et al. (82), Lee et al. (85), Li et al. (88), Liu et al. (93), Liu et al. (94), Magnavita et al. (98), Maraqa et al. (99), Martínez-López et al. (100), Marton et al. (101), Podder et al. (111), Pouralizadeh et al. (113), Rodriguez-Menéndez et al. (116), Si et al. (125), Styra et al. (130), Tselebis et al. (139), Tu et al. (140), Uyaroglu et al. (141), Vagni et al. (142), Xiong et al. (150), Yao et al. (151), Yin et al. (152), Zhang et al. (156)
Female sex	Abdulah and Mohammed (23), Alan et al. (27), Arafa et al. (28), Arshad et al. (29), Azoulay et al. (30), Babore et al. (31), Badahdah et al. (32), Bettinsoli et al. (35), Blekas et al. (36), Bukhari et al. (37), Caillet et al. (40), Chen et al. (46), Chong et al. (13), Civantos et al. (49), Cunill et al. (50), Demirjian et al. (51), Elbay et al. (54), Elkholy et al. (56), Erquicia et al. (57), Giardino et al. (61), Guisti et al. (62), Gupta et al. (65), Han et al. (66), Hasan et al. (67), Holton et al. (69), Hosseinzadeh-Shanjani et al. (71), Hu et al. (72), Huang et al. (73), Jain et al. (75), Jo et al. (77), Juan et al. (78), Lai et al. (84), Lee et al. (85), Liao et al. (90), Liu et al. (93), Magnavita et al. (98), Matsuishi et al. (103), Podder et al. (111), Pouralizadeh et al. (113), Rodriguez-Menéndez et al. (116), Rossi et al. (118), Ruiz-Fernández et al. (119), Sahin et al. (121), Sun et al. (132), Surrati et al. (133), Tam et al. (134), Tang et al. (157), Sagaon-Teyssieret al. (120), Tselebis et al. (139), Uyaroglu et al. (141), Vagni et al. (142), Wang et al. (145), Wilson et al. (147), Yao et al. (151), Yin et al. (152), Youssef et al. (154), Zhang et al. (155), Zhang et al. (156)	Song et al. (127), Liu et al. (95), Veeraraghavan and Srinivasan (143)	Aksoy and Koçak (25), Al Mahyijari et al. (26), Barello et al. (33), Cai et al. (38), Chatterjee et al. (42), Chen et al. (47), Chen et al. (45), Chew et al. (48), Elhadi et al. (55), Elkholy et al. (56), Kim and Choi (81), Kim et al. (82), Lai et al. (84), Leng et al. (86), Li et al. (88), Liu et al. (92), Liu et al. (94), Maraqa et al. (99), Martínez-López et al. (100), Master et al. (102), Mo et al. (105), Que et al. (115), Saricam (122), Shahrour and Dardas (123), Si et al. (125), Styra et al. (130), Sun et al. (132), Tan et al. (135), Wang et al. (146), Xiong et al. (150)
Marital status—married with children	Koh et al. (83), Erquicia et al. (57), Han et al. (66), Holton et al. (69), Hu et al. (72), Saricam (122)	Elbay et al. (54)	Alan et al. (27), Babore et al. (31), Bettinsoli et al. (35), Cai et al. (38), Maraqa et al. (99), Mo et al. (105), Mosheva et al. (106), Pouralizadeh et al. (113), Sun et al. (132), Uyaroglu et al. (141), Xing et al. (149), Yörük and Güler (153)
Marital status—single vs. married	Azoulay et al. (30), Chan and Huak (41), Elbay et al. (54), Gupta et al. (65), Hong et al. (70), Huang et al. (73), Liu et al. (95), Podder et al. (111), Song et al. (127), Sorokin et al. (128), Sun et al. (131), Yao et al. (151), Youssef et al. (154)	Han et al. (66), Jain et al. (75), Li et al. (89), Liu et al. (94)	Babore et al. (31), Badahdah et al. (32), Bettinsoli et al. (35), Cai et al. (38), Chen et al. (47), Chong et al. (13), Elhadi et al. (55), Gupta et al. (65), Hasan et al. (67), Hosseinzadeh-Shanjani et al. (71), Hu et al. (72), Juan et al. (78), Kim and Choi (81), Kim et al. (82), Koh et al. (83), Leng et al. (86), Liao et al. (90), Liu et al. (93),
			Maraqa et al. (99), Master et al. (102), Mo et al. (105), Park et al. (108), Pouralizadeh et al. (113), Ruiz-Fernández et al. (119), Saricam (122), Si et al. (125), Styra et al. (130), Sun et al. (132), Sagaon-Teyssieret al. (120), Tu et al. (140), Uyaroglu et al. (141), Wang et al. (145), Xing et al. (149), Yörük and Güler (153), Zhang et al. (156)
Higher education level	Sun et al. (131), Tan et al. (135), Youssef et al. (154)	Alan et al. (27), Han et al. (66), Hong et al. (70), Ji et al. (76), Kim and Choi (81), Li et al. (89), Liu et al. (94), Xing et al. (149), Yao et al. (151)	Cai et al. (38), Chen et al. (47), Hasan et al. (67), Hosseinzadeh-Shanjani et al. (71), Hu et al. (72), Juan et al. (78), Kim et al. (82), Leng et al. (86), Liu et al. (93) Liu et al. (92), Liu et al. (95), Master et al. (102), Mo et al. (105), Pouralizadeh et al. (113), Si et al. (125), Tu et al. (140), Wang et al. (145), Wang et al. (146), Xiong et al. (150), Yin et al. (152), Yörük and Güler (153), Zhang et al. (156)
**Fixed—Occupational**			
Nurse vs. physician	Alan et al. (27), Barello et al. (33), Bates et al. (34), Bettinsoli et al. (35), Chong et al. (13), Cunill et al. (50), Guisti et al. (62), Goulia et al. (63), Gupta et al. (65), Holton et al. (69), Jo et al. (77), Koh et al. (83), Lai et al. (84), Lee et al. (85), Liu et al. (94), Martínez-López et al. (100), Matsuishi et al. (103), Maunder et al. (104), Nickell et al. (107), Park et al. (109), Phua et al. (110), Prasad et al. (114), Poon et al. (112), Shechter et al. (124), Si et al. (125), Song et al. (127), Tam et al. (134), Tan et al. (135), Tang et al. (157), Vagni et al. (142), Wong et al. (148), Xing et al. (149), Yao et al. (151), Zhang et al. (156)	Chan and Huak (41), Chong et al. (13), Liu et al. (95), Ruiz-Fernández et al. (119), Sorokin et al. (128)	Al Mahyijari et al. (26), Badahdah et al. (32), Cai et al. (38), Caillet et al. (40), Chen et al. (47), Chen, et al. (45), Giardino et al. (61), Juan et al. (78), Liu et al. (93), Maraqa et al. (99), Rodriguez-Menéndez et al. (116), Rossi et al. (118), Sun et al. (132), Surrati et al. (133), Wang et al. (146), Yin et al. (152)
Less work experience	Abdulah and Mohammed (23), Arafa et al. (28), Chatterjee et al. (42), Chong et al. (13), Elbay et al. (54), Elhadi et al. (55), Gupta et al. (65), Holton et al. (69), Li et al. (89), Maunder et al. (14), Song et al. (127), Youssef et al. (154)	Sahin et al. (121), Saricam (122)	Cai et al. (38), Dobson et al. (53), García-Fernández et al. (60), Hu et al. (72), Kim and Choi (81), Kim et al. (82), Koh et al. (83), Leng et al. (86), Liu et al. (94), Maraqa et al. (99), Marton et al. (101), Master et al. (102), Park et al. (108), Pouralizadeh et al. (113), Styra et al. (130), Sun et al. (132), Tselebis et al. (139), Tu et al. (140), Wang et al. (146), Xiong et al. (150), Yörük and Güler (153)
Part-time work status	Nickell et al. (107)		Ruiz-Fernández et al. (119)
**Modifiable—Social**			
Social support—personal		Arafa et al. (28), Babore et al. (31), Cai et al. (39), Cai et al. (38), Chen et al. (44), Guisti et al. (62), Hong et al. (70), Hu et al. (72), Juan et al. (78), Kim and Choi (81), Li et al. (88), Liao et al. (90), Liu et al. (94), Master et al. (102), Song et al. (127), Tam et al. (134), Tselebis et al. (139), Xiao et al. (10), Zhang et al. (156)	
Social support—professional/organizational		Ahmed et al. (24), Arafa et al. (28), Chan and Huak (41), Elbay et al. (54), Fiksenbaum et al. (59), Hong et al. (70), Jung et al. (79), Khattak et al. (80), Li et al. (88), Maraqa et al. (99), Rodriguez-Menéndez et al. (116)	
Adequate information		Chan and Huak (41), García-Fernández et al. (60), Goulia et al. (63), Maraqa et al. (99), Rodriguez-Menéndez et al. (116), Styra et al. (130), Vagni et al. (142)	
Stigma	Elhadi et al. (55), Goulia et al. (63), Juan et al. (78), Koh et al. (83), Master et al. (102), Maunder et al. (104), Maunder et al. (14), Park et al. (108), Park et al. (109), Rodriguez-Menéndez et al. (116), Teksin et al. (138), Verma et al. (144)		
**Modifiable—Psychological**			
Perceived control		Bettinsoli et al. (35), Fauzi et al. (58), Ho et al. (68), Hu et al. (72), Liao et al. (90), Marton et al. (101), Mo et al. (105), Shahrour and Dardas (123), Xiao et al. (10), Xiong et al. (150)	
Loss of control and perceived risk	Son et al. (126), Styra et al. (130), Wong et al. (148)		Hong et al. (70)
Adaptive coping styles and ability		Bettinsoli et al. (35), Chen et al. (47), Chen et al. (45), Fauzi et al. (58), Huffman et al. (74), Lin et al. (91), Master et al. (102), Vagni et al. (142), Wang et al. (145)	Babore et al. (31), Son et al. (126)
Maladaptive coping styles	Babore et al. (31), Chen et al. (47), Chen et al. (45), Lin et al. (91), Master et al. (102), Maunder et al. (14), Phua et al. (110), Wang et al. (145)		
Positive work attitudes		Babore et al. (31), Chan and Huak (41), Goulia et al. (63), Liu et al. (92), Tan et al. (135), Wang et al. (145)	
Adaptive personality traits		Cai et al. (38), Chen et al. (47), Dobson et al. (53), Hu et al. (72), Li et al. (89), Lin et al. (91), Mosheva et al. (106), Park et al. (108), Yörük and Güler (153)	
Maladaptive personality traits	Lu et al. (96), Maunder et al. (14), Yi-Ching et al. (96)		
**Factors related to infection exposure**			
Exposure to confirmed infected cases	Aksoy and Koçak (25), Alan et al. (27), Babore et al. (31), Badahdah et al. (32), Bettinsoli et al. (35), Chen et al. (47), Chen et al. (46), Chong et al. (13), Di Tella et al. (52), Erquicia et al. (57), Fiksenbaum et al. (59), Grace et al. (64), Giardino et al. (61), Guisti et al. (62), Han et al. (66), Hasan et al. (67), Holton et al. (69), Jain et al. (75), Juan et al. (78), Kim and Choi (81), Koh et al. (83), Lai et al. (84), Li et al. (87), Li et al. (89), Liu et al. (92), Liu		Bukhari et al. (37), Maraqa et al. (99), Maunder et al. (14), Sun et al. (132), Tselebis et al. (139), Veeraraghavan and Srinivasan (143), Xiong et al. (150)
	Liu et al. (93), Liu et al. (95), Lu et al. (97), Magnavita et al. (98), Maraqa et al. (99), Maunder et al. (104), Mo et al. (105), Park et al. (109), Poon et al. (112), Rossi et al. (118), Stojanov et al. (129), Sun et al. (131), Surrati et al. (133), Tam et al. (134), Tang et al. (136), Teshome et al. (137), Verma et al. (144), Xing et al. (149), Yin et al. (152), Yörük and Güler (153)		
Increased risk of exposure to confirmed infected cases	Bukhari et al. (37), Caillet et al. (40), Chatterjee et al. (42), Chen et al. (43), Chen et al. (45), Elbay et al. (54), Hu et al. (72), Lee et al. (85), Liao et al. (90), Liu et al. (95), Matsuishi et al. (103), McAlonan et al. (16), Pouralizadeh et al. (113), Ruiz-Fernández et al. (119), Sahin et al. (121), Saricam (122), Si et al. (125), Sorokin et al. (128), Styra et al. (130), Que et al. (115), Wang et al. (146), Yao et al. (151), Zhang et al. (155), Zhang et al. (156)		Dobson et al. (53), Liu et al. (94), Sun et al. (132)
Colleagues being infected, quarantined, deceased	Rossi et al. (118)		
Being in quarantine	Fiksenbaum et al. (59), Liu et al. (92), Maunder et al. (14), Sun et al. (132)		Juan et al. (78), Lee et al. (85), Park et al. (109), Si et al. (125), Zhang et al. (156)
Hospital resources, protection, training		Chen et al. (44), Demirjian et al. (51), Elbay et al. (54), García-Fernández et al. (60), Gupta et al. (65), Hu et al. (72), Huffman et al. (74), Jain et al. (75), Kim and Choi (81), Maraqa et al. (99), Martínez-López et al. (100), Master et al. (102), Mosheva et al. (106), Pouralizadeh et al. (113), Surrati et al. (133), Tam et al. (134), Tang et al. (136), Sagaon-Teyssier et al. (120), Vagni et al. (142)	

Ninety studies tested sex as a possible risk factor for distress among HCW during an outbreak (Table 2), with all but 33 finding that being female was associated with higher risk for psychological distress. Notably, the 57 studies that found that female sex was a significant risk factor spanned six different infectious diseases (MERS, SARS, COVID-19, H1N1, H7N9, and SARS), suggesting that being a female HCW increases vulnerability for distress more generally when working during an infectious outbreak. Notably, among the studies 30 studies that did not find that being female created significant risk for distress, eleven (36.6%) were conducted with nurses and included predominantly female participants (24, 43, 44, 59, 70, 79, 80, 89, 108, 140, 153).

Of the 69 studies that examined marital status as a risk or resilience factor for psychological distress, 19 found evidence to suggest this as a risk factor (Table 2). For example, two studies of HCW during the SARS outbreak found that HCW who were single were 1.4 times more likely to experience psychological distress than married HCW (41), and more likely to have sever depressive symptoms 3 years later (92). Similarly, HCW during the COVID-19 outbreak who were single experienced higher levels of distress than those who were married (54, 57, 66, 69, 111, 122, 126). Conversely, four studies conducted during COVID-19 found that being married was a risk factor for greater distress (66, 75, 89, 94), and two studies found that married HCW with children reported greater stress than single HCW or those who were married without children (72, 83). Forty-seven other studies conducted during the SARS, MERS, and COVID-19 outbreaks found no associations between HCW marital status and distress (Table 2).

Thirty-three studies examined education levels in association with distress. Only eight studies, six conducted during the COVID-19 pandemic (27, 66, 70, 89, 94, 149, 151), along with studies conducted during the Ebola outbreak (76), and the MERS outbreak (81) found that HCW with higher educational levels reported significantly lower psychological distress. Twenty-two studies found that education level was not predictive of psychological distress among HCW working during the MERS or the COVID-19 outbreaks (Table 2).

### Occupational Factors

Thirty-four studies examined and found evidence that the HCW occupational role created risk for psychological distress while working during the SARS, H1N1, MERS, and COVID-19 outbreaks (Table 2). In all but 16 studies, being a nurse was associated with a range of mental health issues, including higher stress, burnout, anxiety, depression, PTSD symptoms, psychiatric morbidity, and psychological distress compared to being a physician or other HCW (see Tables 1, 2). The extent to which nurses experienced greater psychological distress whilst working during an outbreak was estimated in several studies. For example, nurses were 1.2 (83), 1.4 (124), 2.2 (63), and 2.8 (107) times more likely to be at risk for poor mental health. In contrast, five studies found that physicians (13, 97, 119, 128) and technicians (41) were more likely to experience distress while working during the COVID-19 pandemic and the SARS outbreak. Sixteen studies conducted during the COVID-19 outbreak did not find that occupational role was a risk factor for distress (Table 2).

Other occupational factors examined included years of work experience, and full-time vs. part-time status. Twelve of the 35 studies found evidence to suggest that less work experience may create risk (Table 2). HCW who had worked for <2 years experienced significantly greater stress than those with more work experience in a large sample of HCW during the SARS pandemic (13). In HCW during the SARS outbreak, those with <10 years of experience reported higher levels of psychological distress, but not burnout or post-traumatic stress, 13–26 months after the outbreak (14). HCW who had less clinical experience were also more likely to experience stress during the COVID-19 outbreak (23, 28, 55, 65, 69, 154). Years of clinical experience was not associated with PTSD symptoms, acute stress or anxiety, depression, mental health status, or burnout in 21 other studies (Table 2). Two studies found that less work experience was protective against distress for HCW during COVID-19 (121, 122). Lastly, in one study, part-time worker status was a significant predictor of greater emotional distress in HCW during the SARS outbreak (107), whereas another study found no evidence of part-time work status creating risk for distress in HCW during COVID-19 (119).

### Social Factors

A number of social and interpersonal factors mitigated or contributed to psychological distress. Receiving direct social support from friends, family, colleagues and supervisors was a key protective factor in all of the 19 studies that examined its association with psychological distress (Table 2). For example, in HCW during the COVID-19 outbreak, higher levels of social support were associated with significantly lower levels of stress, depression, anxiety, depression and PTSD (28, 31, 38, 62, 70, 78, 88, 90, 94, 102, 139, 156). These findings were consistent with that of a study of frontline medical staff during the COVID-19 outbreak who reported that a positive attitude from co-workers was important for reducing their distress (39). Analogously, emergency nurses working during MERS outbreak who reported poor support from family and friends experienced higher levels of burnout (81). Similarly, studies of HCW during the SARS outbreak found that higher levels of family support were associated with lower depression and anxiety whereas inadequate support from relatives, lack of gratitude from patients and relatives, and perceiving less of a team spirit at work was associated with higher levels of psychological distress (44, 134).

Organizational support was an important factor in buffering psychological distress of HCW during an outbreak in all 11 studies that examined this factor. In nurses working during the SARS outbreak in Canada, higher perceived organizational support in the form of receiving positive performance feedback from doctors and co-workers, was associated with lower perceptions of SARS-related threat and reduced feelings of emotional exhaustion (59). Similarly, nurses, physicians, and HCW working during the MERS, COVID-19, and SARS outbreaks who perceived support from their supervisors and colleagues, experienced better mental health in the form of lower PTSD symptoms, lower distress, and being less likely to develop psychiatric symptoms, respectively (24, 28, 41, 54, 59, 70, 79, 80, 88, 99, 116).

Seven studies examined receiving useful information from others (a common form of social support). In one study, HCW who received adequate communication and information about the H1N1 outbreak from their organization were less likely to experience psychiatric symptoms because it helped them cope better, and worry less about the pandemic (63). Similarly, HCW during the SARS outbreak who had confidence in the information they received from their organization (130), and who received clear communication about directives and how to take precautionary measures (41), experienced reduced psychological distress. HCW working during the COVID-19 outbreak who felt that they did not receive sufficient information, scored significantly higher on anxiety and acute stress than those who were satisfied with the information provided (60, 99, 116, 142).

Negative social perceptions created risk for poor mental health for HCW in all 12 studies that examined this factor. In nurses during the MERS outbreak, perceived social stigma was associated with higher stress and poorer mental health (108). Similarly, during the COVID-19 pandemic, HCW who felt stigmatized, perceived stigma concerning negative public attitudes and disclosing about one's work, experienced higher levels of depression, anxiety, and psychological distress (55, 78, 102, 108, 109, 116, 138). During the SARS outbreak, HCW who felt people avoided their family because of their job were twice as likely to have elevated levels of post-traumatic stress symptoms (83). Importantly, experiencing stigma and avoidance from others was significantly associated with higher levels of post-traumatic stress symptoms during the SARS outbreak (104), and 13–26 months later (14).

### Psychological Factors

The psychological factors examined in the studies included adaptive and maladaptive coping responses, beliefs and attitudes, and personality traits. Fourteen studies examined how perceptions of control were associated with distress among HCW (Table 2). In eight studies, higher self-efficacy was associated with lower anxiety, depression, distress, and lower levels of fear about SARS and post-traumatic stress symptoms during the COVID-19 and SARS outbreaks, respectively (10, 35, 68, 72, 90, 105, 123, 150). Conversely, feeling a loss of control was associated with greater distress (148) during the SARS outbreak in Hong Kong. Analogously, appraisals of personal risk were linked to higher levels of PTSD symptoms in HCW during the MERS (126) and SARS (130) outbreaks. Only one study conducted with nurses during COVID-19 did not find evidence that risk appraisals were linked to greater distress (70).

Positive attitudes toward one's work were protective against distress in all six studies that examined this factor. Higher work satisfaction was associated with less psychological distress among hospital staff during the H1N1 outbreak (63), lower PTSD among nurses (145), and lower rates of burnout among HCW during the COVID-19 outbreak. Similarly, HCW during the SARS outbreak who felt their work had become more important were less likely to develop psychiatric symptoms (41), and those who viewed their work altruistically were less likely to have severe symptoms of depression 3 years later (92). HCW who held a positive attitude toward their work reported less stress during the peak of the COVID-19 outbreak (31).

Seventeen studies examined whether coping styles were associated with HCW distress during an outbreak (Table 2). Emergency physicians and nurses working during the SARS outbreak who used denial, mental disengagement, or venting of emotions to cope were more likely to score higher on psychiatric morbidity (110). Similar results were found in frontline nurses during COVID-19, with use of negative coping associated with higher PTSD and psychological distress (102), and positive coping linked to lower PTSD (145). In HCW during the SARS outbreak, those who used maladaptive coping strategies, such as escape-avoidance, and self-blame coping, reported higher levels of burnout, psychological distress, and post-traumatic stress when surveyed 13–26 months after the outbreak (14). However, the use of adaptive strategies, such as problem-solving and positive reappraisal, were not associated with any of the distress outcomes. This finding was consistent with those from studies in which coping ability was not significantly associated with PTSD symptoms during the MERS outbreak (126), and problem-solving and turning to religion to cope were not associated with reduced distress during COVID-19 (31).

Twelve studies investigated the role of personality in HCW's psychological distress (Table 2). During the SARS outbreak, neuroticism was linked to poorer mental health (96), and HCW who had an anxious attachment style reported experiencing higher burnout, psychological distress, and post-traumatic stress 13–26 months after the outbreak (14). Those with an avoidant attachment style reported greater distress, but not burnout or post-traumatic stress. Eight studies examined the role of dispositional resilience. Among nurses working during the MERS outbreak, higher levels of hardiness were associated with lower stress and better mental health (108), and resilience was associated with lower anxiety, depression, post-traumatic stress symptoms, and burnout among frontline nurses and HCW during COVID-19 (38, 45, 72, 74, 89, 91, 153).

### Factors Related to Infection Exposure

Fifty-three studies examined the impact of direct contact with infected patients on HCW's psychological distress. Of these, the majority (65) found that being in direct contact with and/or treating patients infected with COVID-19, SARS, MERS, or H7N9 was a risk factor for psychological distress (Table 2). Only eight studies did not find that contact with infected patients increased risk for distress in HCW during the COVID-19, SARS, and MERS outbreaks. Similarly, 24 studies found that risk of contact with infected patients due to working in high-risk areas (e.g., ICU, isolation areas and infection units) was associated with higher levels of anxiety, stress, and post-traumatic stress symptoms than not working in such areas (Table 2). Notably, one study found that HCW in a high-risk unit during SARS reported higher and sustained perceived stress 1 year after the outbreak compared to those in low-risk units, with those in low-risk units reporting a decrease in stress over time, but those in high-risk units experiencing an increase in stress post-outbreak (16). Three studies conducted during COVID-19 found that risk of contact was not associated with greater distress (53, 94, 132). Spending time in quarantine due to risk of being infected was associated with higher levels of burnout, depression, and psychological distress in HCW during SARS and COVID-19 (14, 59, 92, 132), but was unrelated to post-traumatic stress symptoms and psychological distress in HCW during the MERS outbreak or the COVID-19 outbreak (78, 85, 109, 125, 156). Lastly, one study found that HCW who had colleagues who became infected, had deceased due to infection, or had been quarantined, also experienced higher levels of post-traumatic stress symptoms and acute stress during the COVID-19 outbreak (118).

Provision of adequate training, protection, and other resources to manage and reduce risk of infection was associated with less psychological distress in all 19 studies that examined this factor (Table 2). Receiving clear infection control guidelines predicted lower psychological morbidity in frontline HCW during SARS (134), and having sufficient hospital resources for the treatment of MERS was associated with lower MERS-related burnout (81). After the implementation of a SARS protection training program, HCW experienced significant decreases in anxiety and depression 2 weeks and 1 month after the starting the program (44). Similarly, medical staff receiving inadequate training related to managing H7N9 had higher PTSD symptoms than those who received appropriate training (81). During COVID-19, HCW who felt HCW who felt that they did not have adequate information, training, personal protective equipment (PPE), felt unsafe, and perceived lower logistic support, reported higher levels of depression, anxiety, and acute stress symptoms (51, 54, 60, 65, 72, 74, 99, 100, 102, 106, 120, 142).

## Discussion

To our knowledge, this rapid systematic review of 139 samples of 143,246 HCW working during an infectious outbreak is the largest and most up to date review of the evidence on the factors that contribute to risk or resilience to psychological distress. In this review we introduced a conceptual framework that categorized the factors contributing to increased and reduced risk of psychological distress among HCW during an infectious disease outbreak into three main categories, including factors that were fixed, modifiable, and related to infection exposure. The majority of the studies reviewed examined the role of fixed factors (demographic and occupational), with fewer studies examining how modifiable factors (social and psychological) were associated with psychological distress in HCW working during an outbreak.

For the fixed factors, the weight of the evidence indicated that HCW who were female or a nurse were at significant risk for psychological distress (Figure 2). Nurses tend to tend to be predominantly female, have higher workloads (104), and have more patient contact than other HCW. Indeed, we found that over 36 percent of the studies that found no significant relationship between being female and increased psychological distress involved only nurses.

There was also clear and consistent evidence that HCW who had or were at risk for contact with infected patients, were more likely to experience psychological distress (Figure 3). Worry about becoming infected is a key stressor for HCW in the context of an outbreak as risk of infection has implications not only for their own health but also for that of their families (83). Evidence also indicated that being in quarantine contributes to distress, perhaps due to being isolated from the team (158), and that vicariously experiencing these risks can be detrimental for HCW mental health (118).

Although relatively fewer studies investigated modifiable factors (Figure 2), the evidence highlighted key target areas to reduce HCW distress. It is also worth noting that the findings from the studies examining the role of social and psychological factors were extremely consistent. This lends confidence to the suggestion that these factors are important targets for intervention to reduce distress and bolster resilience. Stigmatizing attitudes from the public toward HCW were consistently associated with greater distress across the studies reviewed. Although stigma can be effectively reduced through social contact with those who experience stigmatization (159), this approach may not be practical or advisable during an outbreak. Instead, public health campaigns that deliver accurate messages and highlight facts to reduce the fears underlying stigma (160), counteracting the climate of fear cultivated through the media which can promote stigma during an infectious outbreak (161) could assist.

The evidence was unanimous in indicating that perceiving social support was associated with lower distress. Adequate social support is a resilience factor that is well-known to be effective reducing stress across a number of stressful situations (162), and is equally important for reducing stress among HCW (163). This support can come from supervisors and co-workers (164), either formally or informally, through positive performance feedback (59), and positive attitudes, and through peer support groups. Organizational social support may be especially important to fill the gap when personal social support may be sparse because regular social support sources are struggling with their own distress during an outbreak. Such support can also foster positive work attitudes and satisfaction (165), which were associated with lower distress.

The evidence reviewed was also consistent in indicating that harmful coping strategies linked to greater distress, and positive coping strategies were protective for distress. Interventions that target harmful coping strategies, such as avoidance and self-blame, that can that may maintain or increase stress, may be worthwhile. Identifying when HCW may be using such strategies and finding ways to foster more positive approaches for managing stress are important for not only for reducing distress, but also for reducing the risk of other adverse health consequences. For example, HCW who experienced post-traumatic stress during the SARS outbreak and used harmful coping were at greater risk for substance abuse (166). Mental health check-ups are one approach that could help monitor both HCW distress and whether appropriate coping strategies are being used (167).

In keeping with evidence that low perceived control is a transdiagnostic vulnerability factor for anxiety (168), perceptions of control were consistently associated with lower distress in the evidence reviewed. Indeed, having a sense of control is a well-known factor for reducing health-related distress (162). Feeling a loss of control may be inevitable during an infectious outbreak, as perceptions of risk are inversely related to perceived control (169). However, interventions focused on increasing a sense of autonomy can be effective for reducing distress in HCW during times of upheaval (170). The evidence reviewed suggests that this might be accomplished at the organizational level by providing HCW with the resources needed to manage the risk of infection. For example, providing personal protective equipment (PPE), adequate training, and clear guidelines, information, and protocols for infection control are important, because having such resources is linked to lower distress. This conclusion is consistent with research that found that access to information and provision of needed resources increased a sense of empowerment among ICU nurses (171).

Adaptive personality traits consistently linked to better mental health outcomes in HCW working during an outbreak. Dispositional resilience was examined in the majority of the studies reviewed, with hardiness examined in one study. Dispositional resilience can be conceptualized in several different ways, including as a personal quality reflecting the capacity to cope, or as type of hardiness (172). When conceptualized as the former, resilience involves being flexible to change, managing unpleasant emotions, and not getting discouraged (173). Although personality traits are often viewed as being relatively stable, personality can also be viewed as reflecting personal qualities and tendencies that are expressed to a greater or lesser degree, and are therefore amenable to change (174). From this perspective, approaches that help HCW develop a tendency to use resilient coping skills may help reduce vulnerability to psychological distress during an outbreak.

### Limitations and Strengths

There are several limitations of this rapid systematic review. Conducting the review during the ongoing outbreak of COVID-19 imposed time constraints. This meant that we only included published peer-reviewed literature and did not search more thoroughly through gray literature or online pre-print repositories. Most study samples were quite large, increasing confidence in the generalisability of the findings.

In terms of the evidence base, the majority of the studies were cross-sectional, providing only a snapshot of the factors associated with HCW psychological distress. This limits conclusions about the direction of causality between the factors and distress, especially for those factors that are modifiable. Only three studies examined the potential long-term effects of the risk and resilience factors on HCW's mental health by using follow-up and time-lagged designs (14, 16, 92), providing some support for the assumed contribution of the factors to distress. More research needs to track the associations of risk/resilience factors over time with distress and the extent to which certain factors link to sustained or transient distress.

The majority of the studies were conducted during COVID-19, with relatively fewer studies reporting results from other infectious outbreaks such as SARS, MERS, H1N1, H7N9, and Ebola. On the one hand, this could be viewed as a limitation on the generalisability of the findings from the predominant outbreak, COVID-19, to other infectious outbreaks. On the other hand, we would argue that the consistency of the findings for a number of factors including participant sex, being a nurse, all 10 of the social and psychological factors, four of the five infection exposure factors, demonstrate that findings are likely to be generalizable across infectious outbreaks for these factors.

Although a number of studies investigated fixed factors and infection-related factors, relatively fewer studies examined how modifiable factors linked to distress (Figures 2, 3). There is a need for more research focusing on these factors to provide a more solid evidence base about potential targets for clinical intervention and treatment. A handful of studies used unvalidated measures of psychological distress, raising concerns about whether the findings would be the same had validated measures been used. For those studies that used validated measures, the ways in which cut-off scores for caseness were calculated, and/or the ways in classification of symptoms met thresholds for psychological distress, undoubtedly varied between measurement instruments. This likely introduced some variance into the results.

Few studies considered potential confounders in the associations with distress, compared found associations in matched non-HCW samples, or the extent to which the factors were predictive of distress outside of an outbreak. As well, the results extracted from the studies reflect a mix of bivariate and multivariate associations, as not all studies reported the bivariate only findings, which would be more comparable for making comparisons. Studies that examined the factors in multivariate analyses often used different covariates making it difficult to draw equitable conclusions from the studies. It is therefore difficult to assess the degree to which certain factors may independently predict psychological distress over and above other factors. Collectively, these limitations may have contributed to the equivocal findings noted for several of the factors reviewed.

Several strengths of the Review balance these limitations. Conceptually organizing the factors according to risk or resilience and whether they were fixed or modifiable, provided a theoretical framework for identifying who might be at most risk for psychological distress. This facilitates appropriate clinical intervention, and for noting which factors would be suitable targets for potential interventions. We also reported non-significant and contrary findings alongside significant findings to provide a more balanced and critical overview of the evidence. The Review included evidence from across six infectious disease outbreaks, with the majority of the research reporting findings from coronavirus outbreaks—Severe Acute Respiratory Syndrome Coronavirus-2 (COVID-19), Severe Acute Respiratory Syndrome-related coronavirus (SARS), and Middle East Respiratory Syndrome-related coronavirus (MERS)—that share similarities in their symptom and contagion profiles. Consistent evidence for risk and resilience factors was found across these various infectious diseases, suggesting that the findings from this review may be applicable across different outbreaks. This is relevant for understanding the mental health of HCW in future outbreaks. Lastly, conducting a series of search updates ensured integration of the most recent evidence from the ongoing COVID-19 outbreak into the review at the time of submission.

### Implications and Conclusions

Whereas, other reviews have documented the extent of distress experienced by HCW during an outbreak (2), the current Review highlights the profiles of HCW most at risk for psychological distress and psychiatric morbidity during an outbreak. This identified modifiable factors that warrant further investigation as possible points of intervention to mitigate distress. Viewing risk and resilience factors from the lens of fixed and modifiable factors provides an efficient and useful approach for understanding who is most at risk and how to address that risk during and after an outbreak. Further research focusing on possible interactions among these factors would be useful to gain a better understanding of both the risk profiles and key modifiable factors, as the evidence reviewed did not consistently examine this area.

There is evidence that the psychological distress from working during an outbreak can persist for 2–3 years after the outbreak (14–16). Therefore, monitoring and providing appropriate support should continue beyond the outbreak period to ensure mental health recovery, especially among HCW who are most at risk. Our findings suggest that particular attention should be paid to female HCW and nurses (regardless of sex), and those who come into contact with infected patients or their environments to ensure that they receive necessary resources and provision of support to manage psychological distress. Proactive approaches at the organizational level can be effective (164) and may be necessary to help reduce the psychological distress of HCW. For example, a study of HCW during the COVID-19 outbreak in China found that mental health resources and services were mainly used by those experiencing mild and subthreshold levels of psychological distress rather than those who experienced more severe distress (11). Addressing the mental health needs of HCW with more severe distress will likely require more proactive means from health-care organizations.

There are a number of delivery methods to provide support and help HCW modify risk factors and foster resilience factors. These include telehealth, mobile apps, online toolkits, and peer-support, either in person or virtual (175). Combining different approaches may also be effective. For example, social support and perceived control can have an additive effect for reducing stress related to job demands (176). There is also evidence for the effectiveness of interventions for reducing HCW distress when delivered at the person level and organizational level (164), as well as those that target lifestyle practices (177, 178).

Evidence from randomized controlled trials suggests that third-wave cognitive behavioral therapeutic approaches, such as mindfulness (178), gratitude (177), and self-compassion (179), are effective for reducing stress and burnout among healthcare professionals, and could be beneficial. In low-resource settings, peer-support is one option that has been shown to be effective for reducing occupational distress in HCW (164). Raising awareness of the impact of an infectious outbreak on HCW mental health, providing appropriate treatment and therapy, and fostering proactive approaches such as an organizational culture of support (180), are recommended as possible approaches that can help prepare HCW for future outbreaks and address any persistent, long-term distress following the outbreak.

## Data Availability Statement

The original contributions presented in the study are included in the article/Supplementary Material, further inquiries can be directed to the corresponding author/s.

## Author Contributions

FS and JO designed and conceived the study, searched for evidence, screened and analyzed the data, drafted the work, revised it critically for intellectual content, approved the final piece of work for publication, and agree to be accountable for the work. All authors contributed to the article and approved the submitted version.

## Conflict of Interest

The authors declare that the research was conducted in the absence of any commercial or financial relationships that could be construed as a potential conflict of interest.
