# Supplementary material for: Factors Associated With Psychological Distress in Health-Care Workers During an Infectious Disease Outbreak: A Rapid Systematic Review of the Evidence
Source: Front Psychiatry. 2021 Jan 28;11:589545. doi: 10.3389/fpsyt.2020.589545 (PMC7876062; doi:10.3389/fpsyt.2020.589545)
Supplement: Supplementary file 1 [file Data_Sheet_1.PDF]

Supplementary Table 1. Total cumulative global cases of major infectious disease outbreaks

Virus and dates	Cases	Deaths	Recovered	Source
**SARS 1/01/2002-11/07/2003**	8,437	813	7,452	World Health Organisation
**H1N1 2009-2018**	100,500,000	284,000	100,216,000	Centre for Disease Control (CDC)
**H7N9 2013-03/07/20**	1,565	610	955	Centre for Disease Control
**EBOLA 2014-2016**	28,616	11,310	17,306	Centre for Disease Control
**MERS 04/2012-31/01/2020**	2,519	866	1,653	World Health Organisation
**COVID-19 8/01/2020-23/11/2020**	58,751,191	1,389, 770	57,361,421	John Hopkins Coronavirus Resource Centre

NOTE: COVID-19: Severe Acute Respiratory Syndrome Coronavirus-2 (SARS-CoV-2); EBOLA: Ebola virus; H1N1: Influenza A (swine flu); H7N9: Asian lineage avian influenza A; MERS: Middle East Respiratory Syndrome-related coronavirus; SARS: Severe Acute Respiratory Syndrome-related coronavirus
