# Supplementary material for: Factors Associated With Psychological Distress in Health-Care Workers During an Infectious Disease Outbreak: A Rapid Systematic Review of the Evidence
Source: Front Psychiatry. 2021 Jan 28;11:589545. doi: 10.3389/fpsyt.2020.589545 (PMC7876062; doi:10.3389/fpsyt.2020.589545)
Supplement: Supplementary file 2 [file Data_Sheet_2.PDF]

Supplementary Table 2: Quality assessment ratings for the 139 studies.

Study	Rater 1	Rater 2	Agreed Category
1. Abdulah & Mohammed (2020)	10	9	HIGH
2. Ahmed et al. (2020)	10	10	HIGH
3. Aksoy & Kocak (2020)	7	7	MODERATE
4. Al Mahyijari et al. (2020)	10	9	HIGH
5. Alan et al. (2020)	6	8	MODERATE
6. Arafa et al. (2020)	10	9	HIGH
7. Arshad et al. (2020)	10	10	HIGH
8. Azoulay et al. (2020)	11	9	HIGH
9. Babore et al. (2020)	11	9	HIGH
10. Badahdah et al. (2020)	11	10	HIGH
11. Barello et al. (2020)	7	8	MODERATE
12. Bates et al. (2020)	10	9	HIGH
13. Bettinsoli et al. (2020)	9	9	HIGH
14. Blekas et al 2020	11	10	HIGH
15. Bukhari et al. (2016)	7	7	MODERATE
16. Cai, Tu, et al. (2020)	11	9	HIGH
17. Cai, Lian, et al. (2020)	7	7	MODERATE
18. Caillet et al. (2020)	11	10	HIGH
19. Chan & Huak (2004)	8	8	MODERATE
20. Chatterjee et al. (2020)	9	9	HIGH
21. Chen, Wu, et al. (2005)	10	11	HIGH
22. Chen, Chou et al. (2006)	9	9	HIGH
23. Chen, Wang et al. (2020)	9	9	HIGH
24. Chen, Liu. et al. (2020)	10	11	HIGH
25. Chen, Sun, et al. (2020)	11	9	HIGH
26. Chew et al. (2020)	11	10	HIGH
27. Chong et al. (2004)	11	10	HIGH
28. Civantos et al. (2020)	10	10	HIGH
29. Cunill et al. (2020)	11	10	HIGH
30. Demirjian et al. (2020)	11	9	HIGH
31. Di Tella et al. (2020)	10	10	HIGH
32. Dobson et al. (2020)	10	10	HIGH
33. Elbay et al. (2020)	10	10	HIGH
34. Elhadi et al. (2020)	10	9	HIGH
35. Elkholy et al. (2020)	10	11	HIGH
36. Erquicia et al. (2020)	10	11	HIGH
37. Fauzi et al. (2020)	11	11	HIGH
38. Fiskenbaum et al. (2006)	10	10	HIGH
39. García-Fernández et al. (2020)	8	8	MODERATE
40. Giardino et al (2020)	8	8	MODERATE
41. Goulia et al. (2010)	9	9	HIGH
42. Grace et al. (2005)	8	8	MODERATE

43. Guisti et al (2020)	10	10	HIGH
44. Gupta et al. (2020)	8	8	MODERATE
45. Han et al. (2020)	11	10	HIGH
46. Hasan et al. (2020)	8	8	MODERATE
47. Ho et al. (2005) - S1	6	7	MODERATE
48. Ho et al. (2005) - S2	10	10	HIGH
49. Holton et al. (2020)	11	10	HIGH
50. Hong et al. (2020)	10	9	HIGH
51. Hosseinzadeh-Shanjani et al. (2020)	8	8	MODERATE
52. Hu et al. (2020)	11	10	HIGH
53. Huang et al. (2020)	10	11	HIGH
54. Huffman et al. (2020)	10	10	HIGH
55. Jain et al. (2020)	9	9	HIGH
56. Ji et al. (2017)	7	7	MODERATE
57. Jo et al. (2020)	10	10	HIGH
58. Juan et al (2020)	10	10	HIGH
59. Jung et al. (2019)	9	10	HIGH
60. Khattak et al. (2020)	8	6	MODERATE
61. Kim et al. (2016)	10	11	HIGH
62. Kim et al. (2018)	9	10	HIGH
63. Koh et al. (2005)	6	8	MODERATE
64. Lai et al. (2020)	9	11	HIGH
65. Lee et al. (2018)	9	10	HIGH
66. Leng et al. (2020)	10	10	HIGH
67. Li, Chen, et al. (2020)	10	10	HIGH
68. Li, Li, et al. (2020)	8	8	MODERATE
69. Li, Zhou, et a l. (2020)	10	10	HIGH
70. Liao et al. (2020)	11	9	HIGH
71. Lin, et al. (2020)	10	10	HIGH
72. Liu et al. (2012)	9	11	HIGH
73. Liu et al. (2020)	10	10	HIGH
74. Liu, et al. (2021)	11	9	HIGH
75. Liu, Wang, et al. (2020)	11	9	HIGH
76. Lu et al. (2006)	10	10	HIGH
77. Lu et al. (2020)	10	9	HIGH
78. Magnavita et al. (2020)	11	11	HIGH
79. Marqa et al. (2020)	10	9	HIGH
80. Martínez-López et al. (2020)	9	9	HIGH
81. Marton et al. (2020)	8	8	MODERATE
82. Master et al. (2020)	10	10	HIGH
83. Matsuishi et al. (2012)	9	10	HIGH
84. Maunder et al. (2004)	10	10	HIGH
85. Maunder et al. (2006)	10	10	HIGH
86. McAlonan et al. (2007)	9	10	HIGH
87. Mo et al. (2020)	9	10	HIGH
88. Mosheva et al. (2020)	10	10	HIGH

89. Nickell et al. (2004)	9	10	HIGH
90. Park et al. (2018)	10	10	HIGH
91. Park et al. (2020)	9	9	HIGH
92. Phua et al. (2005)	10	10	HIGH
93. Podder et al. (2020)	10	10	HIGH
94. Poon et al. (2004)	11	10	HIGH
95. Pouralizadeh et al. (2020)	9	10	HIGH
96. Prasad et al. (2020)	10	11	HIGH
97. Que et al. (2020)	11	10	HIGH
98. Rodriguez-Menéndez et al. (2020)	9	9	HIGH
99. Romero et al. (2020)	8	8	MODERATE
100. Rossi et al. (2020)	10	10	HIGH
101. Ruiz-Fernández et al. (2020)	9	9	HIGH
102. Sagaon-Teyssier (2020)	9	11	HIGH
103. Şahin et al. (2020)	11	9	HIGH
104. Saricam (2020)	10	10	HIGH
105. Shahrour & Dardas (2020)	10	10	HIGH
106. Shechter et al. (2020)	9	10	HIGH
107. Si et al. (2020)	10	11	HIGH
108. Son et al. (2019)	11	10	HIGH
109. Song et al. (2020)	11	10	HIGH
110. Sorokin et al. (2020)	11	10	HIGH
111. Stojanov et al. (2020)	10	10	HIGH
112. Styra et al. (2008)	10	10	HIGH
113. Sun, Song, et al. (2020)	10	11	HIGH
114. Sun, Yang et al. (2020)	10	9	HIGH
115. Surrati et al. (2020)	9	9	HIGH
116. Tam et al. (2004)	9	10	HIGH
117. Tan et al. (2020)	11	10	HIGH
118. Tang et al. (2017)	10	10	HIGH
119. Teksin et al. (2020)	10	9	HIGH
120. Teshome et al. (2020)	10	11	HIGH
121. Tselebis et al. (2020)	9	10	HIGH
122. Tu et al. (2020)	10	11	HIGH
123. Uyaroglu et al. (2020)	10	10	HIGH
124. Vagni et al. (2020)	8	8	MODERATE
125. Veeraraghavan & Srinivasan (2020)	9	9	HIGH
126. Verma et al. (2004)	9	10	HIGH
127. Wang, Guo, et al. (2020)	10	10	HIGH
128. Wang, Huang., et al. (2020)	11	11	HIGH
129. Wilson et al. (2020)	10	10	HIGH
130. Wong et al. (2005)	9	9	HIGH
131. Xiao et al.(2020)	10	9	HIGH
132. Xing et al. (2020)	9	11	HIGH
133. Xiong et al. (2020)	10	10	HIGH
134. Yao et al. (2020)	8	8	MODERATE

135.	Yin et al. (2020)	10	10	**HIGH**
136.	Yoruk & Guler (2020)	9	10	**HIGH**
137.	Youssef et al. (2020)	10	10	**HIGH**
138.	Zhang, Yang, et al. (2020)	10	10	**HIGH**
139.	Zhang, Shi, et al. (2020)	10	9	**HIGH**

Note: 11 questions with a maximum score of 11; 0-5 = low quality, 6-8= moderate quality, 9-11 = high quality
